# Double sulfur vacancies by lithium tuning enhance CO_2_ electroreduction to n-propanol

**DOI:** 10.1038/s41467-021-21901-1

**Published:** 2021-03-11

**Authors:** Chen Peng, Gan Luo, Junbo Zhang, Menghuan Chen, Zhiqiang Wang, Tsun-Kong Sham, Lijuan Zhang, Yafei Li, Gengfeng Zheng

**Affiliations:** 1 Laboratory of Advanced Materials, Department of Chemistry and Shanghai Key Laboratory of Molecular Catalysis and Innovative Materials, Faculty of Chemistry and Materials Science, Fudan University, Shanghai, 200438 China; 2 Jiangsu Key Laboratory of New Power Batteries, Jiangsu Collaborative Innovation Centre of Biomedical Functional Materials, School of Chemistry and Materials Science, Nanjing Normal University, Nanjing, 210023 China; 3 Department of Chemistry, University of Western Ontario, 1151 Richmond Street, London, ON N6A 5B7 Canada

**Keywords:** Catalytic mechanisms, Electrocatalysis, Nanoscale materials

## Abstract

Electrochemical CO_2_ reduction can produce valuable products with high energy densities but the process is plagued by poor selectivities and low yields. Propanol represents a challenging product to obtain due to the complicated C_3_ forming mechanism that requires both stabilization of *C_2_ intermediates and subsequent C_1_–C_2_ coupling. Herein, density function theory calculations revealed that double sulfur vacancies formed on hexagonal copper sulfide can feature as efficient electrocatalytic centers for stabilizing both CO* and OCCO* dimer, and further CO–OCCO coupling to form C_3_ species, which cannot be realized on CuS with single or no sulfur vacancies. The double sulfur vacancies were then experimentally synthesized by an electrochemical lithium tuning strategy, during which the density of sulfur vacancies was well-tuned by the charge/discharge cycle number. The double sulfur vacancy-rich CuS catalyst exhibited a Faradaic efficiency toward n-propanol of 15.4 ± 1% at −1.05 V versus reversible hydrogen electrode in H-cells, and a high partial current density of 9.9 mA cm^−2^ at −0.85 V in flow-cells, comparable to the best reported electrochemical CO_2_ reduction toward n-propanol. Our work suggests an attractive approach to create anion vacancy pairs as catalytic centers for multi-carbon-products.

## Abstract (web summary)

Electrochemical CO_2_ reduction to the valuable n-propanol is challenging due to the complicated C_3_ forming mechanism. Here, authors demonstrate double sulfur vacancies formed on hexagonal copper sulfide can serve as efficient electrocatalytic centers.

## Introduction

The renewable energy-driven electrochemical carbon dioxide reduction reaction (CO_2_RR) to valuable chemical feedstocks provides a sustainable opportunity to reduce greenhouse gas and store chemical energy^1–6^. Among potential products of electrochemical CO_2_RR, carbon monoxide (CO) and formate (HCOOH) have been routinely achieved^7,8^, with Faradaic efficiencies (FEs) of >95% and partial current densities of well above 100 mA cm^−2^. Several C_1_ and C_2_ deep-reduction (>2e transfer) products^9–11^, including methane (CH_4_), ethylene (C_2_H_4_), and ethanol (CH_3_CH_2_OH), have also been reported with FEs over 50%. However, a C_3_ product, n-propanol (aka. n-PrOH), despite its large energy content and high market price^1,5^, has only been exhibited with limited selectivity (FEs < 10%) and low activity in CO_2_RR^12–15^. The low selectivity of producing n-propanol has been attributed to the challenges of both creating electrocatalytic sites for stabilizing key C_2_ intermediates (*C_2_) to form n-propanol^1,16,17^, and the complicated coupling mechanisms between active C_1_ and C_2_ species, which have previously been suggested with two different modes as CO–CH_2_CHO^15,18,19^ and CO–OCCO^16,20,21^, respectively. OCCOCO* is the most suggested *C_3_ intermediate in CO_2_RR (widely used in theoretical calculations), not only toward n-propanol, but also other products such as acetone^22^. Another mechanism of n-propanol formation was also reported by using CO reaction with acetaldehyde^23^. Recently, the electrochemical CO_2_RR to isopropanol was also reported using carbonized copper metal organic framework-derived electrodes, in which a high reaction temperature (400‒800 °C) was required during the electrocatalysis^24^.

Ion vacancies have been demonstrated as a category of catalytic sites for enhancing CO_2_ activation and *C_1_ adsorption to generate C_≤2_ products^25–27^. For instance, copper oxide with surface oxygen vacancies created by electrochemical reduction showed a ~63% FE of converting CO_2_ into C_2_H_4_^27^. Sargent and coworkers reported that the modification of Cu_2_S core with Cu surface vacancies led to FEs of 8 ± 0.7% for n-propanol^26^. Nonetheless, the negligible selectivity of C_3_ products in those previous works suggests the insufficiency of stabilizing *C_2_ intermediates and further promoting C_1_–C_2_ coupling to generate C_3+_ products. It is reasonable to hypothesize that an effective catalytic site for C_3+_ production should present a high density of negative charges, while conventional approaches of forming vacancies, such as high-temperature annealing^25^ and wet chemical reduction^28^, tend to form dispersed vacancies with limited hierarchical vacancy structures.

**Fig. 1:**
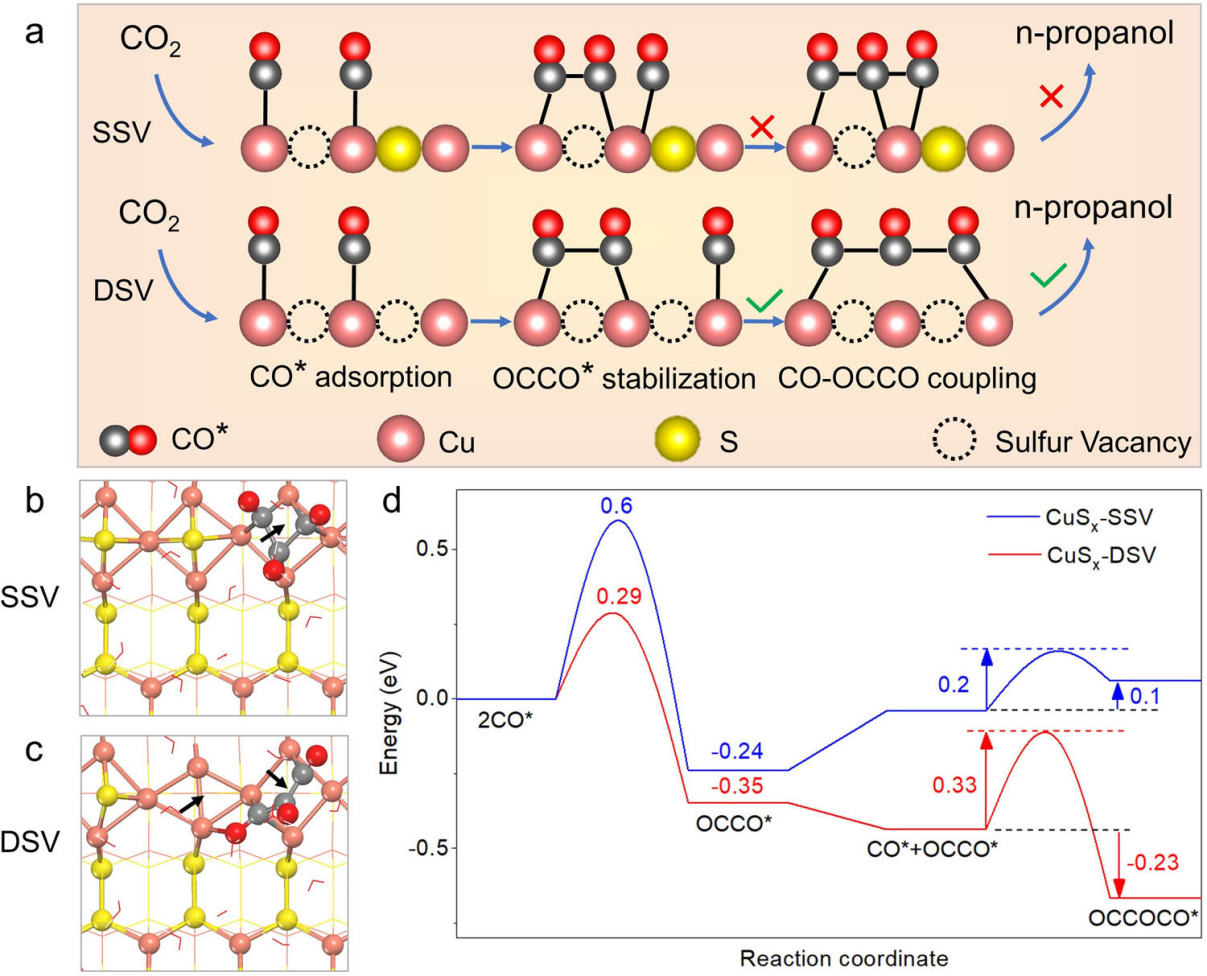
Schematics of CuS_x_ with double sulfur vacancies for converting CO_2_ to n-propanol, and the corresponding calculations. **a** Mechanism of n-propanol formation on adjacent CuS_x_-DSV, showing the dimerization of CO–CO followed by CO–OCCO coupling. **b**, **c** Top views of the optimized OCCOCO* intermediate configurations on (100) surface of **b** CuS_x_-SSV and **c** CuS_x_-DSV. The arrows indicate the positions of sulfur vacancies. **d** Corresponding energy diagrams for CuS_x_-SSV (blue curve) and CuS_x_-DSV (red curve) at 0 V vs. RHE. The pink, yellow, gray, red spheres and red wireframe in **a**–**c** represent copper, sulfur, carbon, oxygen atoms, and water molecules, respectively.

Copper sulfide (CuS) has previously been reported to electrochemically reduce CO_2_ toward major products of HCOOH (FE = ~60%)^29^ or CH_4_ (FE = ~73%)^30^, and its selectivity tuning was ascribed to the catalyst morphologies and the local electronic structures of copper around sulfur atoms. Nevertheless, as a two-dimensional (2D) transition metal dichalcogenides, hexagonal phase CuS possesses abundant surface sulfur atoms along (001) and (100) directions in its layered structure (Supplementary Fig. 1) and relatively low dissociation energy of Cu–S bonds^31,32^. These surface sulfur atoms can be removed to form sulfur vacancies, designated as CuS_x_ (0 < x < 1)^31,33^. The controlled sulfur vacancies can lower the oxidation of copper between 0 and +2, which has been proved to promote C–C coupling^34^. Herein, we developed a double-sulfur vacancy (DSV) structure using a lithium electrochemical tuning approach, which allowed for stabilization of both CO* and a *C_2_ dimer (i.e., OCCO*), as well as subsequent coupling with a third *CO via CO–OCCO (Fig. 1a). The DSV-rich CuS_x_ catalyst exhibited a much-improved FE_n-PrOH_ of 15.4 ± 1% for n-propanol production at −1.05 V versus reversible hydrogen electrode (vs. RHE) in 0.1 M KHCO_3_, corresponding to one of the highest CO_2_RR selectivities to n-propanol reported in H-cells to date. Using a flow cell with 1 M KOH electrolyte, a high partial current density for n-propanol production (j_n-PrOH_) was obtained as 9.9 mA cm^−2^ at −0.85 V vs. RHE without ohmic correction.

## Results and discussion

### DFT calculations of sulfur vacancies

Density functional theory (DFT) calculations were first conducted to investigate the effective vacancy structures as catalytic sites (Methods). CO*, OCCO*, and OCCOCO* are the critical *C_1_, *C_2_ and *C_3_ intermediates adsorbed on Cu atoms, respectively, and the energy barrier and energy change are used to assess the capability of C–C coupling. The adsorption energies of those intermediates were first calculated on hexagonal CuS(001) surfaces (Supplementary Fig. 2 and Supplementary Table 1). It is found that no matter zero, single, double, triple, or quadruple sulfur vacancies exist, the distance between two neighboring Cu atoms around sulfur vacancies is still relatively far (>3 Å), thus not capable of inducing coupling of multiple adsorbed CO* toward *C_2_ or *C_3_ intermediates.

Unlike (001) planes, the CuS(100) surface presents closer distance between neighboring Cu atoms, due to the shorter interlayered S–S distance (<3 Å) (Supplementary Fig. 1). As the rate-limiting steps for n-propanol formation^16,20,21^, the coupling energies of two CO* (to form an OCCO* dimer) and between the OCCO* dimer and a third CO* (to form an OCCOCO* trimer) were first calculated, respectively. Our results show that although a single sulfur vacancy (SSV) on CuS(100) can dimerize two CO* into OCCO* with an adsorption energy decrease of –0.24 eV (Supplementary Fig. 3 and Supplementary Table 2), the addition of a third CO* destabilizes the system with an increased energy of 0.1 eV (Fig. 1b, d). The reason why SSV cannot achieve the CO–OCCO coupling is the lack of adjacent sulfur vacancy centers, and thus cannot accommodate the strong electrostatic repulsion of three CO* that are all adsorbed at a single sulfur vacancy center.

In contrast, the DSV on CuS(100) allow to enrich negative charges near two adjacent co-planar vacancies (Supplementary Fig. 4), and can stabilize the OCCO* dimer with an adsorption energy decrease of –0.35 eV (Supplementary Fig. 5 and Supplementary Table 2). Moreover, it also enables further coupling with a third CO* to form a polyline OC–COCO trimer, with a decreased energy of –0.23 eV (Fig. 1c, d). Thus, the reason that DSV-rich CuS_x_(100) site can stabilize OCCO* and promote CO–OCCO coupling is attributed to the synergetic effect of two adjacent sulfur vacancies (i.e., DSVs), including the enriched negative charge density to adsorb three CO*, close distance of between adjacent Cu atoms (<3 Å) to couple CO–CO, and suitable space to relax the concentrated charges of OCCOCO* (Supplementary Table 3).

### Lithium electrochemical tuning for sulfur vacancies

CuS nanocrystals were synthesized by hydrolysis of CuCl_2_·2H_2_O and thioacetamide (C_2_H_5_NS) at 60 °C (Methods)^35^. The as-made product presented a nanoflower morphology assembled by 2D nanosheets (Supplementary Fig. 6a). X-ray diffraction (XRD, Supplementary Fig. 6b) analysis revealed a hexagonal phase with a space group of P63/mmc; *a* = *b* = 3.7938 Å, *c* = 16.3410 Å; *α* = *β* = 90°, *γ* = 120° (JCPDS# 03-065-3588). The high-angle annular dark-field scanning transmission electron microscopy (HAADF-STEM) mapping further confirmed the existence and uniform distribution of Cu and S elements in CuS nanosheets (Supplementary Fig. 7). The lattice spacing of 2D nanosheets was 0.281 nm (Supplementary Fig. 8), corresponding to the (103) planes of hexagonal CuS, consistent with the XRD result.

**Fig. 2:**
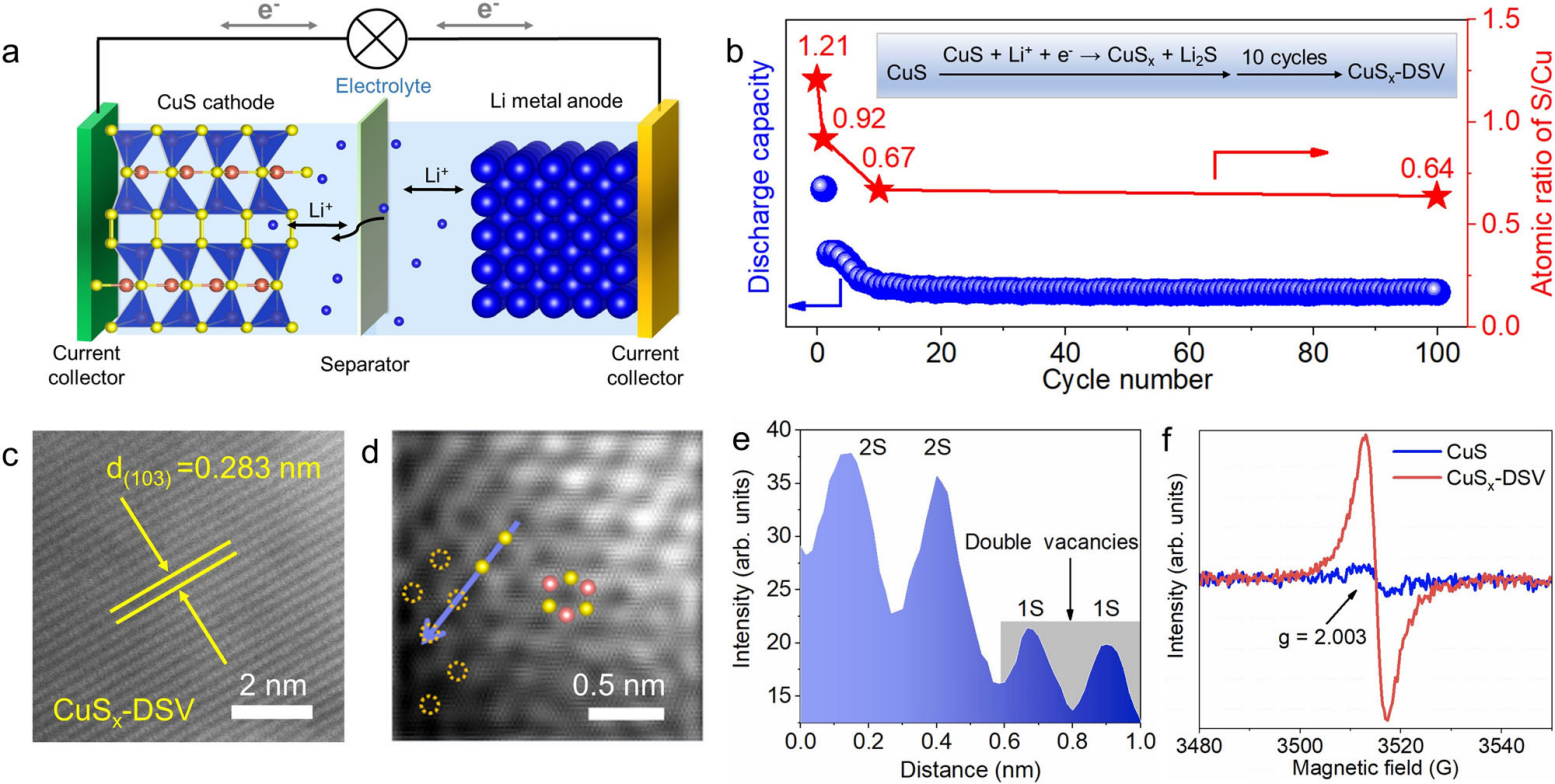
Lithium electrochemical tuning method and structural characterizations. **a** Schematic diagram of lithium-ion battery assembled with CuS (cathode) and Li metal (anode). **b** Discharge capacity of CuS (blue dots) and the atomic ratio of S/Cu (red stars) with respect to the cycle number at a constant current of 0.044 mA·cm^-2^ in the voltage range of 0.01–3 V. Sulfur atoms in the CuS lattice were controllably taken away to form Li_2_S, resulting in CuS_x_-DSV. **c** Spherical aberration corrected HRTEM, **d** HAADF-STEM image, and **e** corresponding intensity profiles extracted from the blue line of CuS_x_-DSV in **d**. The typical lattice arrangement of hexagonal phase CuS was presented by the higher contrast of Cu and the lower contrast of sulfur atoms. The intensity of lower left sulfur atoms was around 20 units, almost half of S atoms (~ 40 unit), suggesting the absence of single sulfur atoms. The pink, yellow spheres, and yellow dashed circles indicate copper, sulfur atoms, and sulfur vacancies, respectively. **f** ESR spectra of CuS (blue curve) and CuS_x_-DSV (red curve).

Afterward, the sulfur vacancy-rich CuS_x_ was synthesized by an electrochemical lithium tuning strategy (Fig. 2a), in which the as-made CuS and a Li foil were used as the cathode and anode materials, respectively (Methods). Based on the partial conversion reaction of CuS + Li^+^ + e^−^ → CuS_x_ + Li_2_S (0 < x < 1), Li^+^ was intercalated into the CuS lattice to form Li_2_S and sulfur vacancies. The sulfur vacancy concentration was tuned by the charge/discharge cycle numbers, including 1, 10, and 100 cycles, whereas the corresponding samples were labeled as CuS_x_-1-cycle, CuS_x_-10-cycle, and CuS_x_-100-cycle, respectively. The HAADF-STEM images and corresponding mapping of all the obtained CuS_x_ showed a uniform distribution of both Cu and S elements (Supplementary Fig. 9−11). The atomic ratio of S/Cu in CuS_x_ was determined by energy-dispersive spectroscopy (EDS), which reduced from original 1.21 to 0.92 (for cycle number of 1), 0.67 (for cycle number of 10), and 0.64 (for cycle number of 100), (Fig. 2b, Supplementary Table 4), indicating the continuous removal of sulfur atoms and formation of sulfur vacancies. The charge/discharge curves versus time showed the decreased discharge capacity with increased cycle numbers (Supplementary Fig. 12), suggesting more and more sulfur atoms were removed by lithium ions.

### Structure characterizations

The HAADF-STEM images showed that the spacing of CuS_x_-10-cycle nanosheets was 0.283 nm (Fig. 2c), corresponding the CuS(103) facets. The (103) planes on the edge of CuS_x_-1-cycle and CuS_x_-100-cycle were also observed (Supplementary Fig. 13). Spherical aberration corrected HAADF-STEM was conducted to investigate the detailed structure of sulfur vacancies. The CuS_x_-10-cycle nanosheets exhibited the typical hexagonal phase, where Cu and S atoms were displayed with high and low contrasts, respectively (Fig. 2d). DSV sites were observed at the edge of the nanosheets (highlighted as yellow dashed circles in Fig. 2d). The extracted intensities of two adjacent single sulfur vacancies were about half of two sulfur atoms (Fig. 2e), confirming the existence of double vacancies^28,36^. Furthermore, the electron spin resonance (ESR) spectra of the as-prepared CuS nanosheets did not present any unpaired electrons (Fig. 2f). In contrast, for CuS_x_-DSV, a pair of opposite peaks were clearly observed with a signal at *g* = 2.003, suggesting the existence of unpaired electrons due to the DSV^25,28^.

**Fig. 3:**
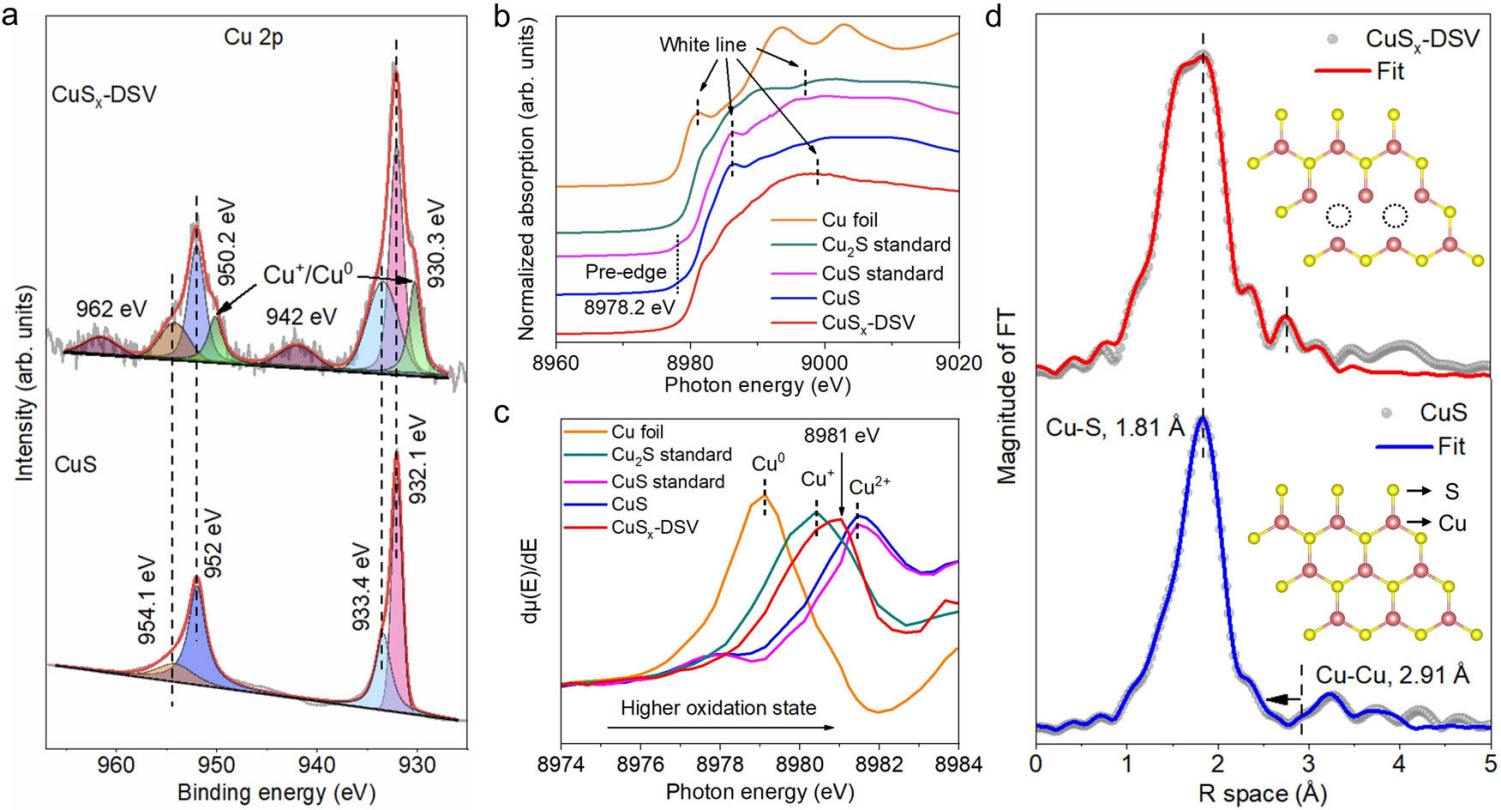
Electronic and fine structural characterizations. **a** XPS spectra of CuS (lower panel) and CuS_x_-DSV (higher panel). **b**, **c** Normalized Cu *K*-edge XANES and the first derivative *μ(E)/dE* of CuS, CuS_x_-DSV, Cu foil, standard CuS, and Cu_2_S samples. **d** Fourier-transformed *k*^*2*^*χ(k)* of CuS (lower panel) and CuS_x_-DSV (upper panel). The insets in **d** are (100) facets on CuS without (lower inset) or with (upper inset) adjacent double sulfur vacancies. The black dashed cycles represent an adjacent sulfur vacancy pair. The decreased coordination number and scattering Cu–S path length (the extracted fitting results in Supplementary Table 4) suggest the absence of sulfur atoms around copper.

X-ray photoelectron spectroscopy (XPS) was conducted to study the oxidation states of Cu element on the catalyst surfaces (Fig. 3a). For the CuS nanosheets, two main peaks around 931.9 and 951.8 eV were observed, corresponding to Cu 2*p*_3/2_ and Cu 2*p*_1/2_ peaks, respectively. The two peaks were further deconvoluted into four sub-peaks, assigned to Cu^2+^ 2*p*_3/2_ (932.1 eV), Cu^2+^ 2*p*_3/2_ (933.4 eV), Cu^2+^ 2*p*_1/2_ (952 eV) and Cu^2+^ 2*p*_1/2_ (954.1 eV), respectively^26^. The peaks located at 933.4 and 954.1 eV were attributed to slight surface oxidation of CuS in air^35^. For CuS_x_-DSV, a new pair of shoulder peaks located at 930.3 and 950.2 eV were observed, corresponding to Cu^+^ or Cu^0^ 2*p*_3/2_/2*p*_1/2_ peaks^12^. The appearance of Cu^+^ was attributed to the partial reduction of Cu^2+^ in CuS. The peaks centered around 942 and 962 eV were ascribed to satellite peaks of Cu^+^ or Cu^0^, respectively^12^.

X-ray absorption spectroscopy (XAS) was conducted to investigate the electronic structure of CuS_x_. X-ray Absorption Near-edge Fine Structure (XANES) spectroscopy of Cu *K*-edge in CuS nanosheets showed a pre-edge feature around 8978.2 eV (Fig. 3b), similar to that of standard crystalline CuS powder^37^. However, compared to standard Cu_2_S and CuS, CuS_x_-DSV displayed a different shape of the rising edge and the post edge between 8990 and 9020 eV (Fig. 3b), suggesting their different chemical valance states and local coordination environments. In addition, compared to standard crystalline Cu powder, CuS_x_-DSV did not present any similar Cu^0^ feature based on the absorption edge or the location of white lines. The first derivatives of the normalized *μ(E)* spectra, *dμ(E)/dE*, were further extracted to highlight the difference (Fig. 3c). The energy value of the first large peak, *E*_*0*_, was defined as the value of absorption threshold or the lowest energy state reached by the core electron excitations^38^. The *E*_*0*_ value of CuS_x_-DSV was 8981.0 eV, which was located between standard Cu_2_S (8980.4 eV) and CuS (8981.4 eV), further indicating the co-existence of Cu^+^ and Cu^2+^ species.

Compared to the CuS nanosheets, CuS_x_-DSV showed a decreased intensity in *k*-space (Supplementary Fig. 14), indicating its lower coordination number (CN) and relatively higher disorder^39^. Fourier-transformed *k*^*2*^*χ(k)* of Cu *K*-edge Extended X-ray Absorption Fine Structure (EXAFS) spectra were further conducted to quantitatively assess the CN and bond length information of these samples (Fig. 3d), which can be used to investigate the local chemical environment of copper around double sulfur vacancies^40^. The strong peak at *R* = 1.81 and small envelope peak around *R* = 2.91 were assigned to the Cu–S and Cu–Cu scattering paths (without phase shifts correction) in the first shell^37^. After fitting, compared with the CuS nanosheets, the CN of the first shell Cu–S path in CuS_x_-DSV decreased from 3 to 1.0 ± 0.2 (Supplementary Table 5), suggesting the formation of abundant sulfur vacancies around the Cu centers^40^. According to the coodination number of Cu–S path and the ratio of sulfur vacancies/total sulfur atoms, the concentration of double sulfur vacancies was calculated as 2.78%, similar to the value of 2.53% calculated in our theoretical model of DSVs (Supplementary Table 6). The length of Cu–Cu path decreased from 3.23 ± 0.03 Å to 2.78 ± 0.02 Å, ascribed to the closer local distance between Cu atoms due to sulfur vacancies. The results were consistent with the optimized CuS_x_-DSV model in our DFT calculations, where the average length of Cu–Cu around DSV sites was below 3 Å, substantially shorter than the theoretical value of 3.23 Å in hexagonal CuS crystal (Supplementary Table 3 and Supplementary Table 5).

### Electrochemical CO_2_ reduction

**Fig. 4:**
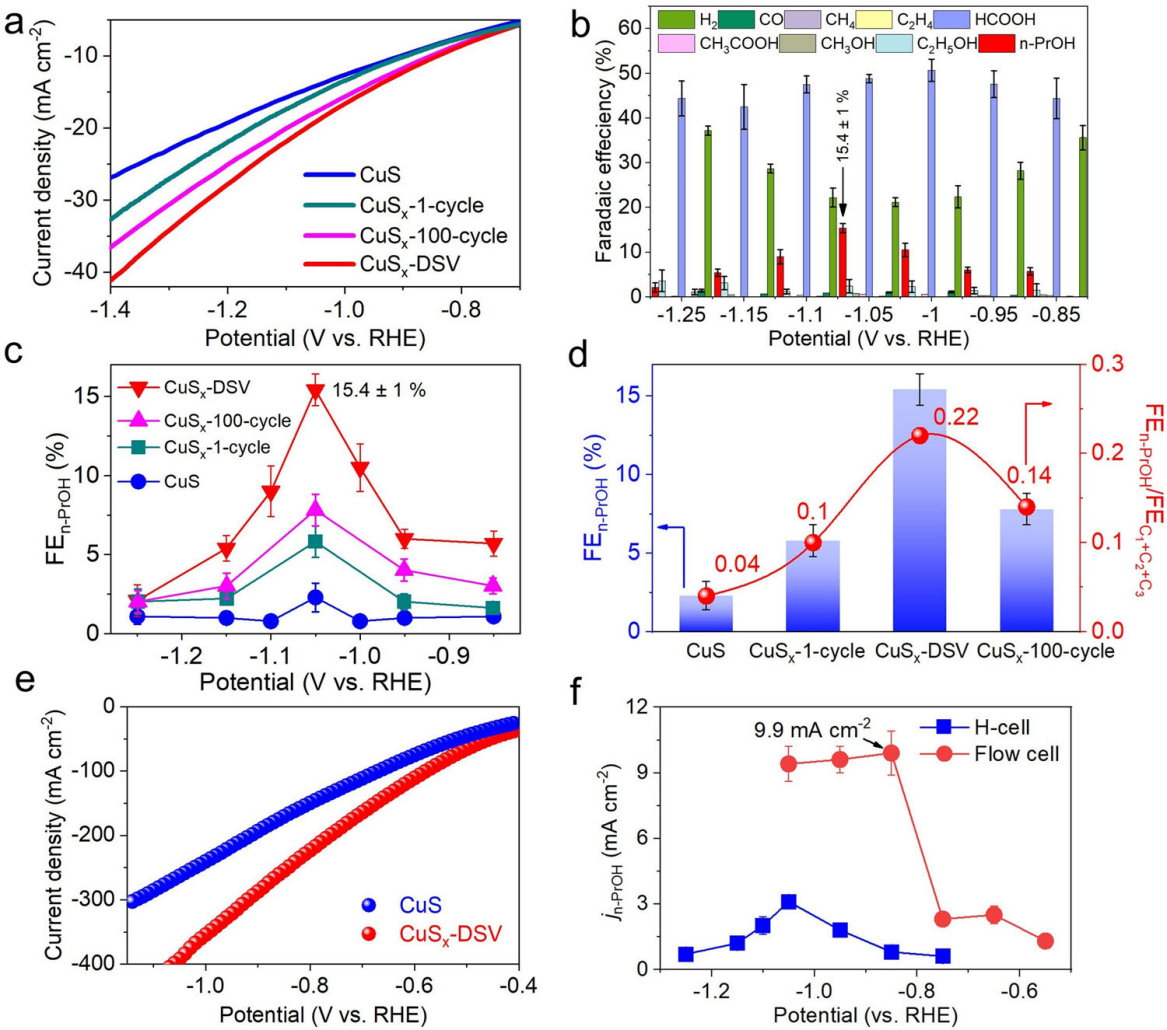
Electrocatalytic CO_2_RR in H-cells and flow-cells. **a** Linear sweep voltammetry curves of CuS, CuS_x_-1-cycle, CuS_x_-DSV, and CuS_x_-100-cycle catalysts in CO_2_-saturated 0.1 M KHCO_3_ aqueous electrolyte with the scan rate at 50 mV s^‒1^. **b** CO_2_RR product distribution using CuS_x_-DSV catalysts in H-cells. **c** FE of n-propanol on the four catalysts at different applied potentials. **d** FE_n-PrOH_ and the ratio FE_n-PrOH_/FE_C1+C2+C3_ of the four catalysts at −1.05 V vs. RHE. **e** Linear sweep voltammetry curves of CuS and CuS_x_-DSV catalysts in 1 M KOH aqueous electrolyte in flow cells. **f** Partial current densities of n-propanol versus potentials using CuS_x_-DSV catalyst in H-cells (blue curve) and flow-cells (red curve). The data in **a**–**d** and **e**–**f** were tested in H-cells and flow-cells, respectively. Error bars in **b**–**d** and **f** correspond to mean ± standard deviation from three measurements.

Based on the aforementioned hypothesis that the DSV-rich CuS_x_ can stabilize OCCO* intermediates and further enhance the CO–OCCO coupling to generate n-propanol, the electrocatalytic CO_2_RR was first conducted using CuS_x_-DSV as the cathode catalyst in a CO_2_-saturated 0.1 M KHCO_3_ aqueous solution under ambient conditions (Methods in the Supplementary Information). Compared with those of the CuS nanosheets, the CuS_x_-1-cycle and CuS_x_-100-cycle catalysts, the CuS_x_-DSV catalyst presented the largest total current density in the linear sweep voltammetry (LSV) curves (Fig. 4a), suggesting its highest electrocatalytic activity. The gas and liquid products were determined by gas chromatography (GC) and ^1^H nuclear magnetic resonance (^1^H-NMR), respectively. Both CuS and CuS_x_-DSV catalysts presented a major product selectivity of HCOOH in the potential window between –0.85 and –1.25 V vs. RHE (Fig. 4b, Supplementary Fig. 15, Supplementary Table 7, 8). Importantly, the ^1^H-NMR spectra of liquid products of CuS_x_-DSV showed a clear triplet at a chemical shift around 0.77 (Supplementary Fig. 16), corresponding to the methyl hydrogen of n-propanol^20^. The CuS_x_-DSV catalyst exhibited a clearly enhanced selectivity of n-propanol, with a peak FE_n-PrOH_ of 15.4 ± 1% and a corresponding partial current density (*j*_n-PrOH_) of 3.1 ± 0.2 mA cm^−2^ at −1.05 V vs. RHE (Fig. 4c). This *j*_n-PrOH_ value was enhanced for almost 10 times compared with that of the CuS catalyst without sulfur vacancies (0.32 ± 0.14 mA cm^−2^). In addition, the gas chromatography–mass spectrometry (GC–MS) spectra showed the fragment ions matched well with the standard database of n-propanol (Supplementary Fig. 17).

To further confirm the n-propanol formation, ^13^CO_2_ isotope labeling experiment was also conducted, and the products were analyzed by ^1^H-NMR. Compared with the original triplet peak at 0.77 and 0.83 ppm with ^12^CO_2_ as feedstock, another two weak peaks were observed around 0.64 and 0.84 ppm when using ^13^CO_2_ as feedstock^23^, as well as the doublet at 8.08 and 8.57 ppm (Supplementary Fig. 18a), indicating the existence of –^13^CH_3_ group of n-propanol and –^13^COOH group on formate^41^. The chemical shift centered at 1.03 and 1.43 corresponded to –^13^CH_2_– group on n-propanol and –^13^CH_3_ group on ethanol, respectively (Supplementary Fig. 18b)^23^. The relative low selectivity to C_2_H_5_OH (FE ~ 4%) for CuS_x_-DSV catalyst was ascribed to the consumption of *C_2_ intermediates by further C_1_–C_2_ coupling to form n-propanol, similar to the reported electrochemical results^14,42^.

By contrast, the CuS catalyst showed negligible selectivity toward n-propanol (2.3 ± 0.9%, Supplementary Fig. 15), as well as other possible products including CO, CH_4_, C_2_H_4_, CH_3_OH, CH_3_COOH (all below 1%). The CuS_x_-1-cycle catalyst showed a higher FE_n-PrOH_ than CuS, but lower than CuS_x_-DSV (Supplementary Fig. 19), which was ascribed to the lower concentrations of DSV sites resulted from insufficient conversion reaction with Li^+^. By tuning the cycle number, the selectivity of n-propanol (i.e., the percentage in all CO_2_RR products, C_1_ + C_2_ + C_3_) was tuned from 4% to 22% with a 5.5-fold increase (Fig. 4d). However, when more cycles of electrochemical lithium tuning were applied, i.e., 100 cycles, both the FE_n-PrOH_ and FE_n-PrOH_/FE_(C1+C2+C3)_ values dropped down, which was ascribed to the damage of the formed DSV sites (Supplementary Fig. 20).

In addition, after 10 h of continuous electrochemical CO_2_RR test at −1.05 V vs. RHE, the FE_n-PrOH_ of CuS_x_-DSV was maintained at 12.1%, corresponding to ~ 78% retention (Supplementary Fig. 21), suggesting its excellent stability. After CO_2_RR, the CuS_x_-DSV catalyst still preserved a similar morphology as that before electrochemical measurement (Supplementary Fig. 22). The Cu *K*-edge XANES spectra (Supplementary Fig. 23a, b) showed that the position of the white line was 8981.8 eV, higher than the Cu foil of 8981.3 eV. The *μ(E)/dE* spectra showed its *E*_*0*_ (8980.9 eV) was downshifted about 0.1 eV, suggesting a slight reduction of copper. Fourier-transformed *k*^*2*^*χ(k)* (Supplementary Fig. 23c, d) showed that the relative intensity ratio between Cu–Cu and Cu–S paths were slightly increased compared to that before CO_2_RR, suggesting that more sulfur atoms were taken away during the CO_2_RR test. After fitting, the coordination numbers of Cu–Cu and Cu–S paths were 4.2 and 0.6, respectively (Supplementary Table 9). The double sulfur vacancies were calculated as 3.89%.

As HCOOH is the major product on all those CuS and CuS_x_ catalysts, DFT calculations were further conducted to study the relationship between HCOOH, CO and n-propanol products. On Cu-based electrocatalysts, *OCHO and *HCOOH are the widely accepted intermediates to produce HCOOH^43^, while *COOH and CO* are the intermediates toward CO^43^. First, it was found that all CuS(100), CuS_x_-SSV(100), and CuS_x_-DSV(100) surface have relatively lower free energy barriers to form *HCOOH (Supplementary Figs. 24 and 26a) than CO* (Supplementary Figs. 25 and 26b), in accord with our electrochemical CO_2_RR results that HCOOH is the main product. Second, for the CO* formation pathway, the desorption of CO molecule is spontaneous on both CuS and CuS_x_-SSV surfaces, but nonspontaneous on CuS_x_-DSV surface (Supplementary Fig. 26b), which allows for subsequent CO–CO coupling. Our calculations indicate that the formation pathways of *HCOOH and CO* are competing with each other, and the existence of sulfur vacancies helps to enhance the probability of forming CO* on catalyst surface. Combined with our aforementioned calculations of CO–OCCO dimerization in Fig. 1d, it can be concluded that CuS_x_-DSV allowed to produce n-propanol via both CO* and OCCO* intermediates, much more significant than on either CuS or CuS_x_-SSV.

In order to promote the yield of n-propanol, the electrochemical CO_2_RR of the CuS_x_-DSV catalyst was further conducted in flow-cells with 1 M KOH aqueous electrolyte. Due to its greatly enhanced mass transport than that in H-cells^10^, the total current density was significantly increased from 20 to 390 mA cm^−2^ at the same potential of –1.05 V vs. RHE (Fig. 4e), indicating an almost 20-fold enhancement. A larger peak partial current density (*j*_n-PrOH_) of 9.9 ± 1 mA·cm^−2^ was achieved at an even lower applied potential of –0.85 V vs. RHE (Fig. 4f, Supplementary Fig. 27, Supplementary Table 10, 11), comparable to the best reported results for n-propanol production (Supplementary Fig. 28 and Supplementary Table 12)^12–15,26,42,44,45^. After 4500 s continuous electrolysis test at –0.85 V vs. RHE, the FE_n-PrOH_ of CuS_x_-DSV was maintained at 3.1%, corresponding to about 80% performance retention (Supplementary Fig. 29).

Finally, to exclude the influence of poly(vinylidene fluoride) (PVDF) binder using in the lithium tuning process, PVDF was prepared as the electrode to perform CO_2_RR test, as a control. In either H-cells or flow cells, H_2_ was the major product with corresponding FE values over 85% in the whole range of voltage tested (Supplementary Fig. 30), while the total FEs of all carbon products were below 5 − 10%. These control experiments confirmed that PVDF had no catalytic selectivity to convert CO_2_ into n-propanol.

In summary, we have demonstrated that double sulfur vacancies formed in hexagonal CuS(100) planes can feature as active electrocatalytic centers for CO_2_RR, enabling the stabilization of CO* and OCCO* dimer, and further coupling CO–OCCO to form the key *C_3_ intermediate of n-propanol. Then, we developed a facile lithium electrochemical tuning approach to enable the formation of the high density of double sulfur vacancies and the decrease of Cu–Cu distance. The FE of n-propanol production was enhanced to 15.4% in H-cells, and the partial current density of n-propanol production was further increased to 9.9 mA cm^−2^ in flow cells, comparable to the best reported values for electrochemical CO_2_RR toward n-propanol. Our work suggests an attractive strategy of applying the lithium electrochemical tuning method to achieve a host of new structures with tailorable ion vacancies as active electrocatalytic sites.

## Methods

### Synthesis of CuS nanosheets

In a typical synthesis, 0.36 g of CuCl_2_·2H_2_O was dissolved in the mixed solution of 13 mL of ethanol and 26 mL of deionized (DI) water to form a green transparent solution A, and 0.18 g of thioacetamide (C_2_H_5_NS) was dissolved in the mixed solution of 10 mL of ethanol and 20 mL of DI water to form a colorless transparent solution B. Afterwards, the solution B was added dropwise into solution A with slow stirring to form a yellow suspension, which was immediately kept in 60 °C water bath for 24 h. Then, the mixture was cooled down to room temperature and washed with DI water and ethanol, each for three times, followed by drying in 60 °C vacuum oven overnight. The black powder of CuS was then obtained.

### Synthesis of vacancy-rich CuS_x_

For introducing sulfur vacancies, the as-made CuS powder was mixed with poly(vinylidene fluoride) with a weight ratio of 7:1, and dispersed in N-methyl pyrrolidone (NMP) under ultrasound to form a black suspension. Afterwards, it was coated uniformly on the surface of a fresh copper foil and dried at 80 °C for about 4 h to remove the solvent. Next, a lithium-ion battery device, using the CuS as the cathode, a lithium foil as the anode, and 1 M LiPF_6_ in ethylene carbonate/dimethyl carbonate/ethyl methyl carbonate (1:1:1 vol%) as the electrolyte, was assembled in CR2016-type coin cells in a glovebox filled with pure argon gas (O_2_ < 1 ppm and H_2_O < 1 ppm). A current density of 0.044 mA cm^−2^ was applied in the voltage window of 0.01–3 V vs. Li^+^/Li. After 1, 10, and 100 cycle(s) of charge and discharge, the CuS cathode was dissembled, rinsed with water and then acetone for several hours to remove the electrolyte and Li_2_S by-product. The solvent of NMP was volatilized at 80 °C. The CuS_x_ catalysts with different density of sulfur vacancies were then purified and obtained.

## Supplementary information

Supplementary Information

## Data Availability

The data that support the findings of this study are available from the corresponding author upon reasonable request.
