# Supplementary material for: Double sulfur vacancies by lithium tuning enhance CO2 electroreduction to n-propanol
Source: Nat Commun. 2021 Mar 11;12:1580. doi: 10.1038/s41467-021-21901-1 (PMC7952561; doi:10.1038/s41467-021-21901-1)
Supplement: Supplementary file 1 — Supplementary Information [file 41467_2021_21901_MOESM1_ESM.pdf]

Supplementary Information

Double Sulfur Vacancies by Lithium Tuning Enhance CO_2 Electroreduction to n-Propanol

Chen Peng,[1] Gan Luo,[2] Junbo Zhang,[1] Menghuan Chen,[1] Zhiqiang Wang,[3] Tsun-Kong Sham,[3] Lijuan Zhang,[1] Yafei Li,[2,*] and Gengfeng Zheng[1,*]

[1]Laboratory of Advanced Materials, Department of Chemistry and Shanghai Key Laboratory of Molecular Catalysis and Innovative Materials, Faculty of Chemistry and Materials Science, Fudan University, Shanghai 200438, China.

[2]Jiangsu Key Laboratory of New Power Batteries, Jiangsu Collaborative Innovation Centre of Biomedical Functional Materials, School of Chemistry and Materials Science, Nanjing Normal University, Nanjing 210023, China

[3]Department of Chemistry, University of Western Ontario, 1151 Richmond Street, London, ON N6A 5B7, Canada.

*Address correspondence to: gfzheng@fudan.edu.cn (G.Z.), and liyafei@njnu.edu.cn (Y.L.)

Supplementary Methods

Chemicals and materials.

$CuCl_2 \cdot 2H_2O$ (≥99.0%), thioacetamide (≥99.0%), $KHCO_3$ (≥99.5%), KOH (≥85%), ethanol (≥99.7%), acetone (≥99.5%), were all purchased from Sinopharm Chemical Reagent Co., Ltd. Commercial CuS and Cu_2S powders were purchased from Aladdin Reagent (Shanghai) Co., Ltd. The impedance of deionized (DI) water was 18.2 $M\Omega \cdot cm^{-1}$.

Characterizations.

The XRD data were characterized by Bruker SMART APEX (II)-CCD (Germany). X-ray photoelectron spectroscopy was recorded on a Perkin-Elmer PHI 5000C ESCA system (Perkin Elmer, USA). The SEM images were performed using a JEM 2100F (JEOL, Japan). The TEM, HRTEM, EDS analysis and HAADF-mapping were performed using a Titan Cubed Themis G2 300 (FEI) microscope. The spherical aberration corrected HRTEM and HAADF-STEM images were collected on the FEI titan Themis Z. ^{1}H-NMR measurements were performed on a Bruker NMR600. ESR spectra was performed on a Bruker ESR A300 spectrometer at room temperature. GC-MS data was collected on the Agilent facility constructed by the 7697A headspace sampler, 7890B GC system and 5977B MSD. XANES and EXAFS data were carried out on 20BM beamline at Advanced Photon Source (APS, Argonne national laboratory, USA). Athena and Artemis software included in a standard IFEFFIT package were used to process XAS data.

Electrochemical measurements.

In a typical synthesis, 5 mg of catalyst was dispersed in 1 mL of acetone, followed by the addition of 120 μL of 5 wt% Nafion solution (Dupont). The mixture was sonicated thoroughly to

form a homogeneous ink. A two-compartment electrochemical H-cell was used with a proton exchange membrane (Nafion 117, Dupont) as the separator and 0.1 M $KHCO_3$ (pH 6.8) as the electrolytes. The working electrodes were prepared by drop-casting the catalyst ink onto carbon paper to achieve a loading density of ~ 1.0 mg·cm^{-2}. Before the measurements, the cathode was purged with pure Ar for 30 min. Flow-cells were constructed using IrO_2 as the anode and an anion exchange membrane as the separator. The working electrodes were prepared by depositing 15 mg of catalyst mixed with 360 µL of Nafion in 3 mL acetone on a carbon gas-diffusion layer (GDL) substrate using air-brush. The CO_2 flow rate in H-cell and flow-cell were both kept at 30 standard cubic centimeters per minute (sccm). The 1 M KOH electrolyte flow was kept at 10 mL·min^{-1}.

All the electrochemical performance measurements were performed using an CHI660D electrochemical workstation (CH Instrument Inc.) at room temperature. In the three-electrode system, saturated calomel electrode (SCE) in H-cell and Ag/AgCl electrode in flow-cell were used as reference electrodes, Pt wire was used as counter electrodes. All potentials were converted to the reversible hydrogen electrode (RHE) scale according to:

$$E \text{ (vs. RHE)} = E \text{ (vs. SCE)} +0.242 \text{ V}+0.0591\times\text{pH} = E \text{ (vs. Ag/AgCl)} +0.197 \text{ V}+0.0591\times\text{pH} \quad (1)$$

Determination of CO_2 reduction products.

Gas products were collected and analyzed by in-line gas chromatograph (Shanghai Ramiin GC 2060). A thermal conductivity detector (TCD) and a flame ionization detector (FID) were used to quantify H_2, CO and other alkane contents, respectively. Liquid products were quantified using fresh sample in 10% D_2O with dimethyl sulfoxide (DMSO) as an internal standard by ^{1}H-NMR spectroscopy (Bruker AVANCEAV III HD 500) via a water suppression mode.

The Faradaic efficiency (*FE*) of the liquid products can be calculated as:

$$FE = \frac{n \times F \times p \times V_{gas}}{i \times R \times T} \times 100\ \% = \frac{n \times c \times V_{liquid} \times F}{Q} \times 100\ \% \quad (2)$$

where n is the number of transferred electrons, F is the Faraday constant, p = 101.3 kPa, V_{gas} is the volume of gas products, i is the total current detected by the electrochemical workstation, R is the gas constant, c is the molar concentration, V_{liquid} is the volume of anode electrolyte, and Q is the quantity of applied electric charges during the CO_2 reduction. The FE data in H-cell were obtained after the electrolysis at 20 mA for the accumulated charge of ~ 30 C, corresponding to the electrolysis time of 1500 s. The electrolysis in flow cells at 200 mA for the accumulated charge of ~ 60 C, corresponding to the electrolysis time of 300 s.

The partial current densities (j) for products can be calculated as below, where A is the geometric area of the cathode:

$$j = \frac{i \times FE}{A} \quad (3)$$

Computational details.

Based on density functional theory (DFT), structural optimization and energy calculations were performed within GGA-PBE[1] functional as implemented in *Vienna ab-initio* Simulation Package (VASP)[2]. The projector augmented wave (PAW) method was used to treat exchange-correlation energy. A plane wave expansion with kinetic-energy cutoff of 450 eV and a residual force tolerance of 0.03 eV/Å were adopted in all calculations to reach sufficiently precise results. The optimized cell parameters of hexagonal CuS structure are a = b = 3.81 Å and c = 16.51 Å, which is in good agreement with experimental measurement. On the basis of experimentally crystalline characterization, we built (001) and (100) facets of CuS with 3×3×1 and 3×1×1 slabs, respectively, where only the top two layers of atoms were allowed to relax. Accordingly, a 3×3×1

and a 3×2×1 Monkhorst-Pack *k*-point meshes were used to sample the k-space, respectively. The last number is the supercell number of unit cell in direction of crystal plane. All slab models are at least consisting of four Cu-S-Cu atom layers for CuS (100) facet, while it is three Cu-S-Cu atom layers for CuS (001) facet. A 20 Å of vacuum space in *z*-direction was selected to remove the effect of slab interactions. To decouple the electrostatic interaction for asymmetric slabs, a dipole correction was included in all cases[3, 4]. Additionally, one layer of water molecules and an extra hydrogen atom were placed onto the slab surfaces to explicitly take solvation and electric filed effects into account[4]. Plane-averaged electrostatic potential for a CuS (100) slab with one water layer on one side is shown in **Supplementary Fig. 31**. The long-range dispersion interactions were described by the D3 correction method[5]. The geometries and barriers of transition state for the CO* coupling process were calculated by climbing-image nudged elastic band (CI-NEB) method[6]. By employing computing hydrogen electrode (CHE) model, the free energy correction can be written as:

$$G = E + ZPE - TS$$

where E is the electronic energy, ZPE is the zero-point energy, and S is entropy. The values of these parameters were obtained from vibrational frequency calculations using harmonic approximation for adsorbates and standard thermodynamics tables for gas phase molecules. Since the values of free energy corrections are nearly the same for one adsorbate on different sulfur vacancy sites, energy and free energy change will have little difference for CO* coupling process. Thus, we used energy change to represent the energy barrier of C–C coupling. The charge density difference is defined as $\Delta\rho = \rho^{100\text{-vac}} + \rho^{S} - \rho^{100}$, where $\rho^{100\text{-vac}}$ and ρ^{100} refers to the charge density of (100) facet with and without S vacancy, respectively. The iso-surface value for both single and double vacancies is 0.0018 e bohr^{-3}. ρ^{S} is the charge density of the removed S atoms.

Supplementary Figures

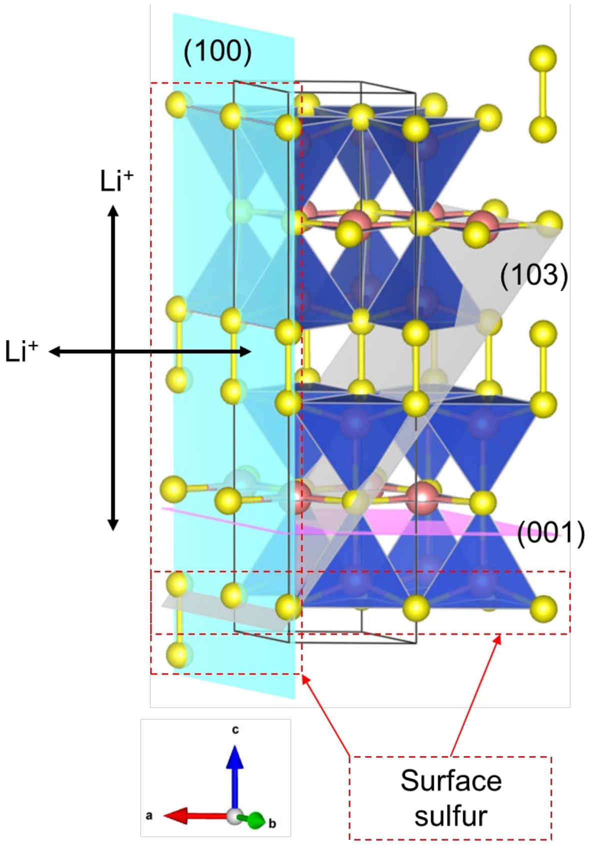

Supplementary Fig. 1 Hexagonal CuS was constructed through the two centrosymmetric sandwich structures, exposing the high-density S atoms on the base (100) (light blue rectangle) or (001) (pink rectangle) crystal planes, and low-density S atoms on (103) planes (gray rectangle). The pink and yellow spheres represent Cu and S atoms, respectively. The solid black lines represent a single cell. The surface sulfur atoms are enclosed by the red dashed line. The two solid line represent the probable migration direction of lithium in the CuS host.

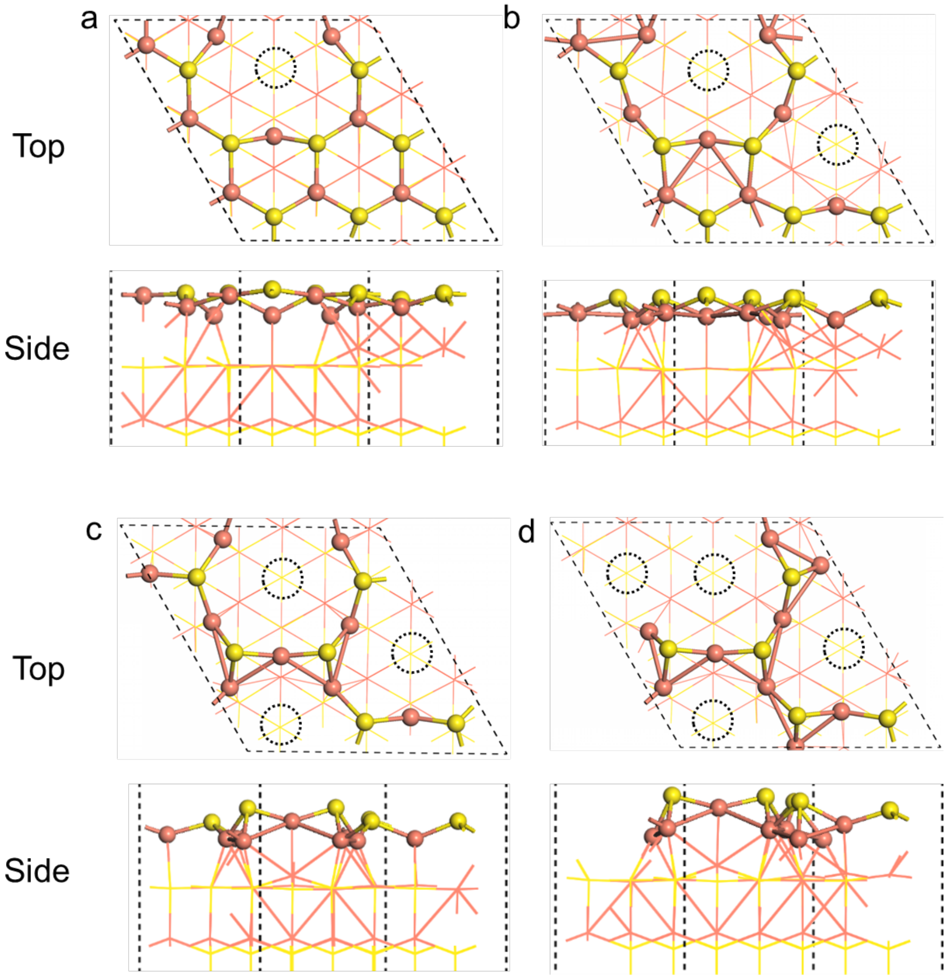

Supplementary Fig. 2 Top and side view of four types sulfur vacancy models on (001) facets after optimization. a Single sulfur vacancy**, b** Double sulfur vacancy**, c** Triple sulfur vacancy and **d** Quaduaple sulfur vacancy. The black dashed cycle represents the sulfur vacancy. The concentration of sulfur vacancies is calculated to be 2.8%, 5.6%, 8.3% to 11.1% based on the ratio of sulfur vacancies/total sulfur atoms in the model. The pink, yellow, gray, red, white spheres, and red wireframes in **a**–**c** represent copper, sulfur, carbon, oxygen, hydrogen atoms, and water molecules, respectively.

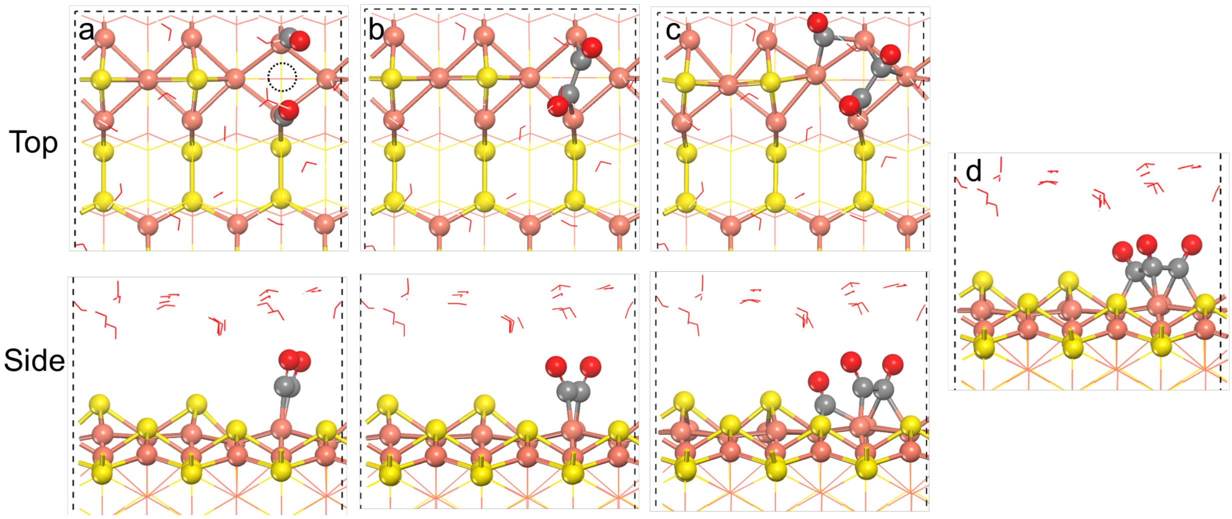

Supplementary Fig. 3 Top view and side view of the single sulfur vacancy (SSV) models on CuS(100) facets after optimization. a Two CO* are adsorbed on the top of two Cu atoms with the sulfur vacancy at the center; **b** dimerization of the two CO* to form OCCO*; **c** the third CO* is adsorbed on the two neighboring bridge Cu atoms. **d** Side-view of the CO–OCCO dimerization. The black dashed circle in **a** represents the single sulfur vacancy.

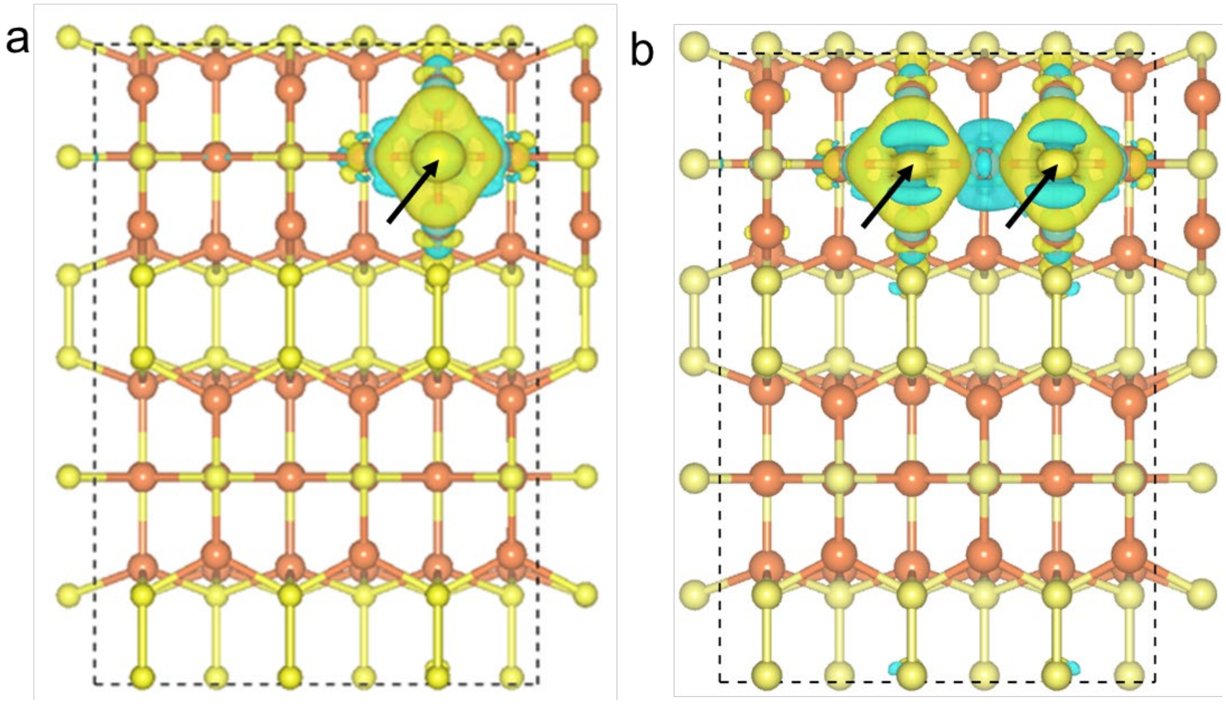

Supplementary Fig. 4 The crystal structure with charge density contour plots of the adjacent double sulfur vacancy-rich $CuS_x(100)$, showing the negative charge enrichment on the Cu atoms near the sulfur vacancies. The double sulfur vacancies (DSV) have one more active site than single sulfur vacancy (SSV), indicating the stronger constrain to the $*C_2$ intermediates. The light yellow and cyan-blue area represent the enrichment of negative and positive charges, respectively. The arrows in **a** and **b** indicate the positions of sulfur vacancies. The enriched negative charge in **b** were induced by the coplanar two sulfur vacancies, leading to the below neighboring seven bivalent Cu^{2+} in the scheme diagram of **Supplementary Table 3** will show the lower oxidation. The lower oxidation than +2 of four copper around the single vacancy in **a** is similar to the situation in **b**.

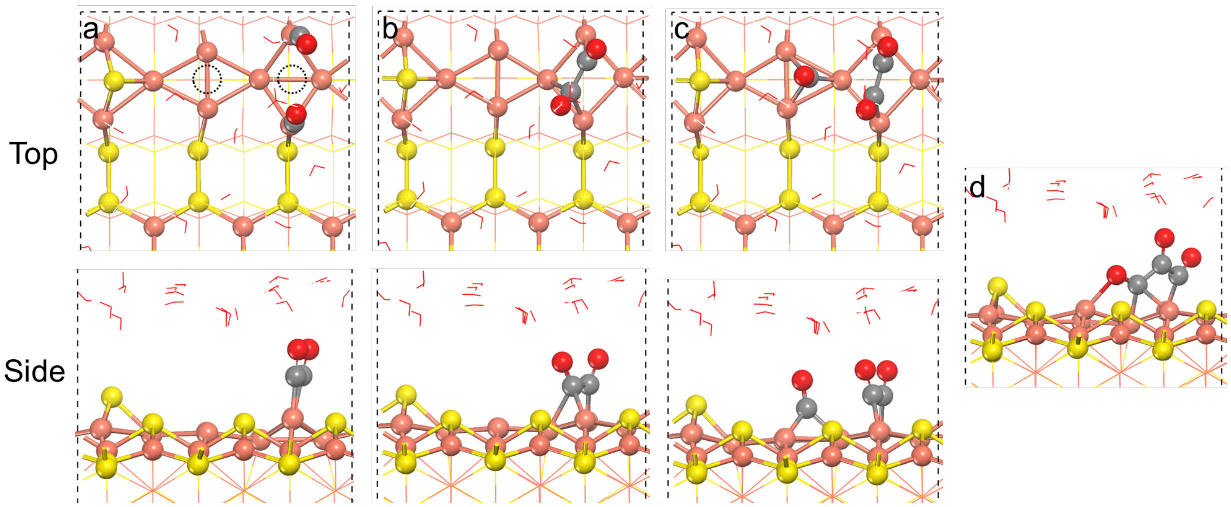

Supplementary Fig. 5 Top view and side view of the double sulfur vacancies (DSV) models on CuS(100) facets after optimization. a Two CO* are adsorbed on the top of two Cu atoms; **b** dimerization of the two CO* to form OCCO*; **c** the third CO* is adsorbed on the third neighboring bridge Cu atom. **d** Side-view of the CO–OCCO dimerization. The black dashed circles represent the double sulfur vacancies.

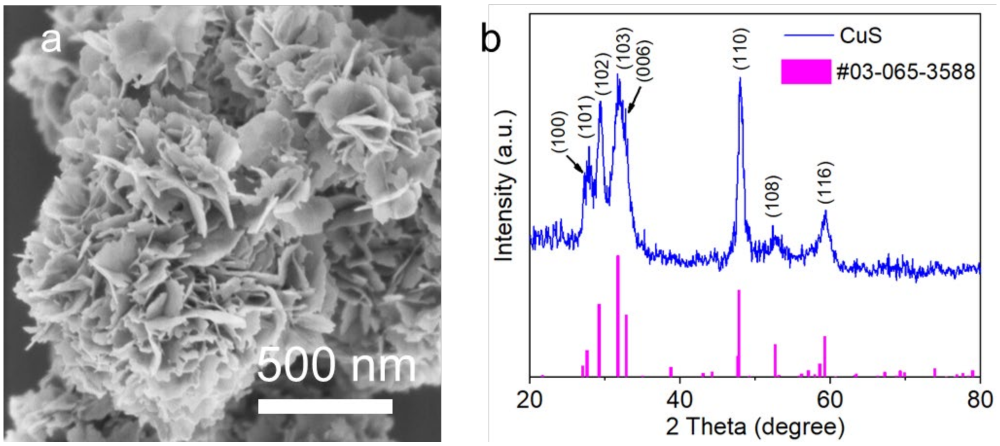

Supplementary Fig. 6 a SEM images and **b** XRD spectrum of CuS.

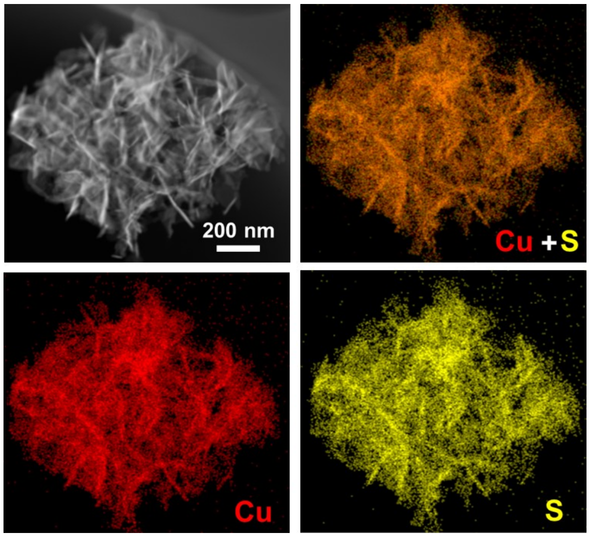

Supplementary Fig. 7 The HAADF-STEM image and the corresponding mapping of CuS.

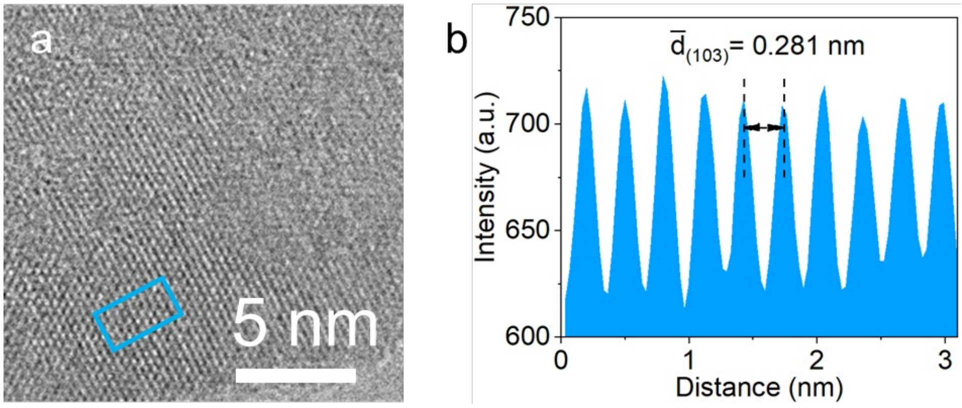

Supplementary Fig. 8 a The HRTEM image and **b** its extracted intensity file of CuS.

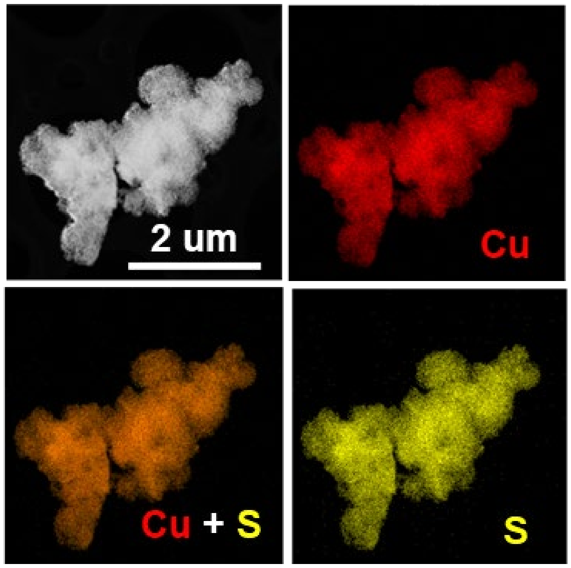

Supplementary Fig. 9 The HAADF-STEM image and the corresponding mapping of CuS_x-1-cycle.

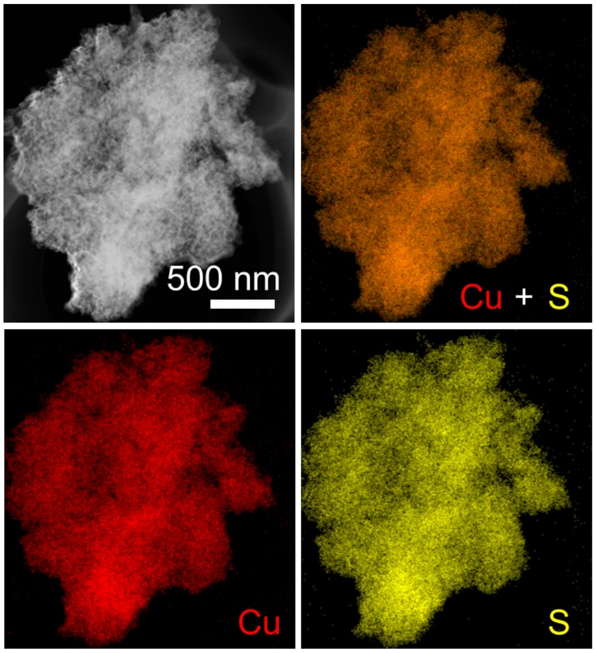

Supplementary Fig. 10 The HAADF-STEM images, and the corresponding mapping of CuS_x-DSV.

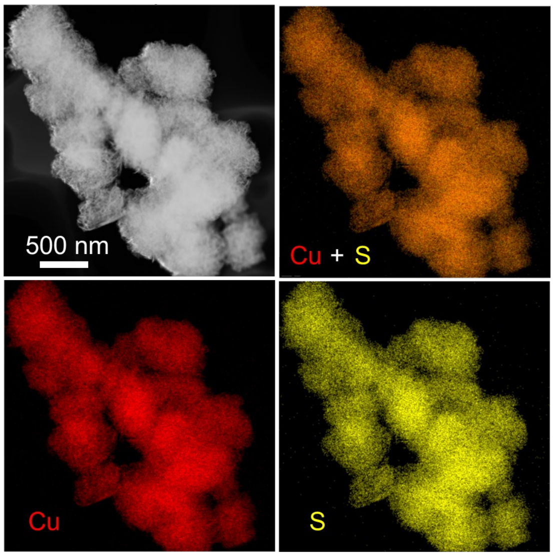

Supplementary Fig. 11 The HAADF-STEM images, and the corresponding mapping of CuS_x-100-cycle.

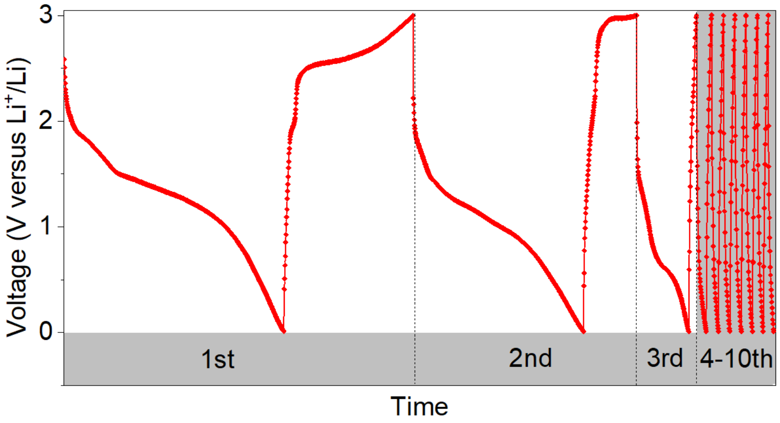

Supplementary Fig. 12 The charge/discharge curves of CuS as the electro material in the voltage window from 0.01 to 3 V vs. Li^+/Li with the current density of ~0.044 mA cm^{-2} in lithium ion battery.

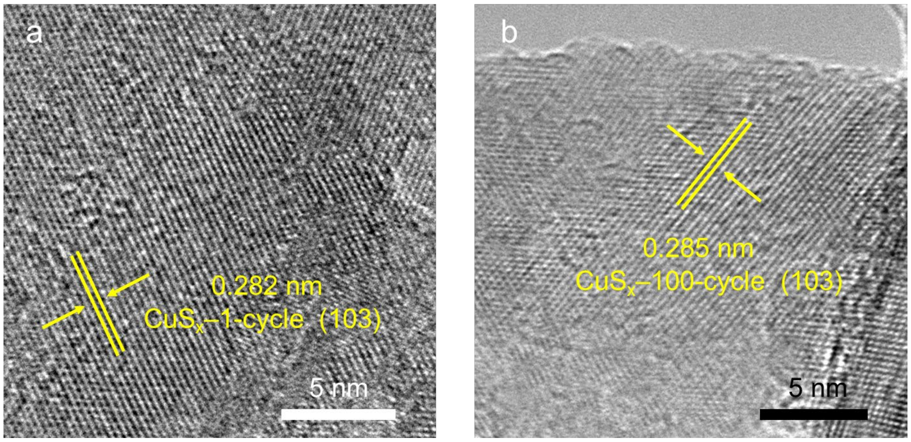

Supplementary Fig. 13 The HRTEM images of **a** CuS_x-1-cycle and **b** CuS_x-100-cycle.

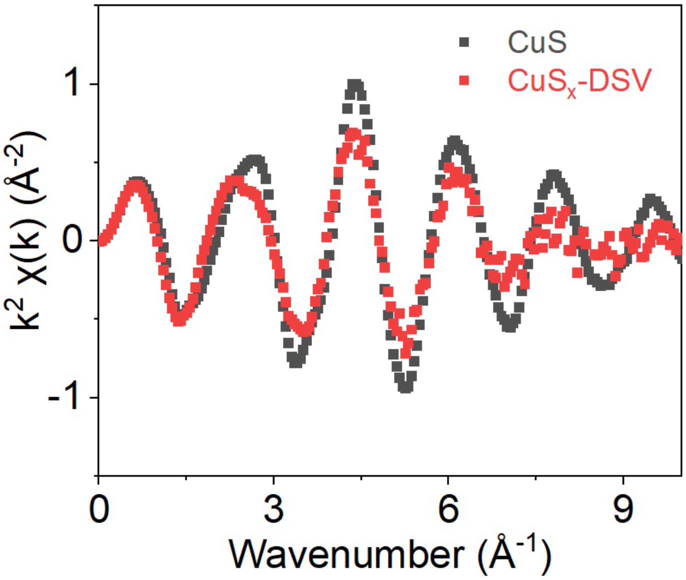

Supplementary Fig. 14 K-space of CuS (black curve) and CuS_x-DSV (red curve).

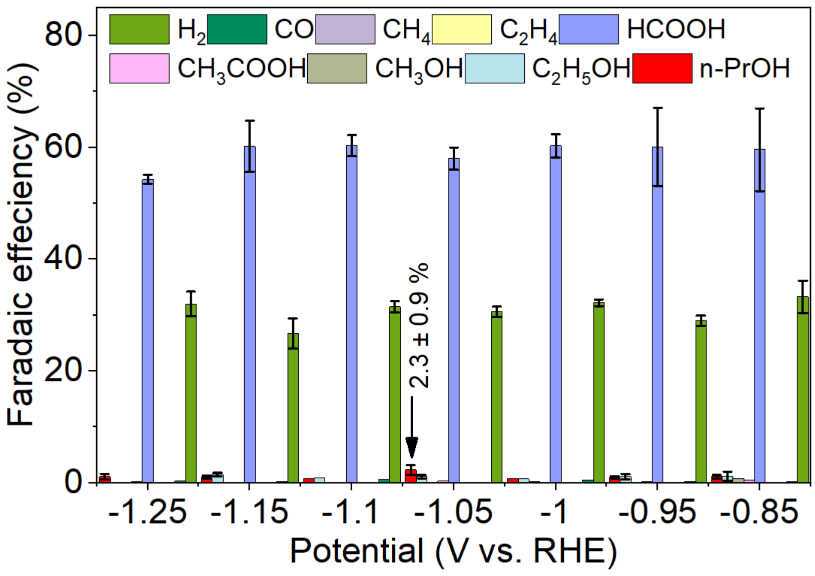

Supplementary Fig. 15 CO_2RR products distribution using CuS catalysts in H-cells. Error bars correspond to mean ± standard deviation at three measurements.

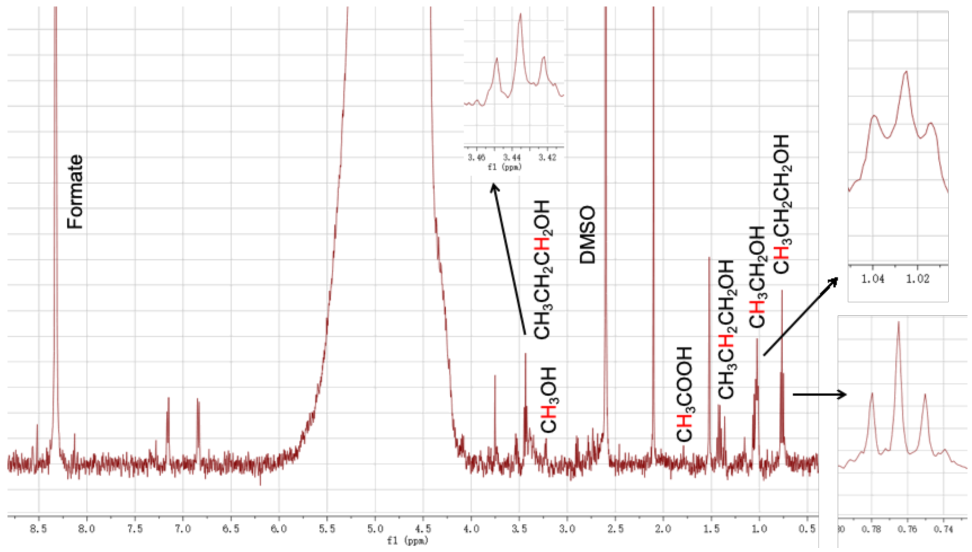

Supplementary Fig. 16 ^{1}H NMR spectra of CuS_x-DSV at –1.05 V vs. RHE after CO_2RR in H-cells.

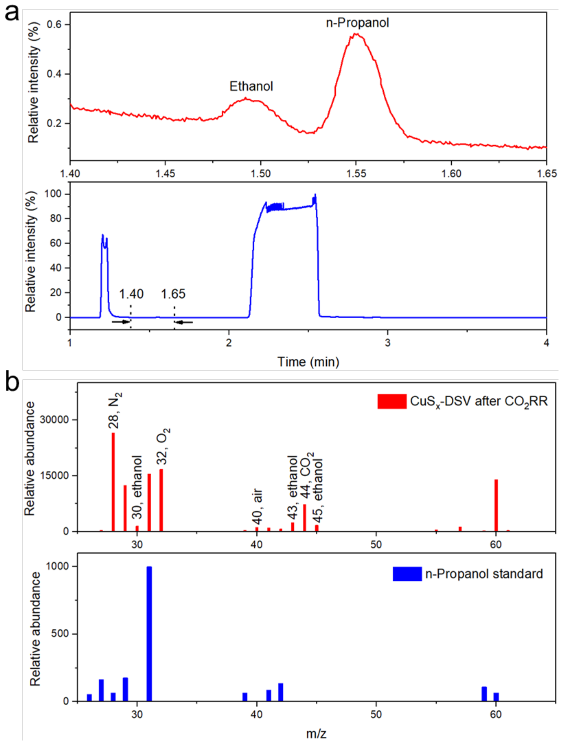

Supplementary Fig. 17 a Gas chromatograph and **b** Mass spectra extracted from the peaks centered at the time around 1.55 min using CuS_x-DSV catalyst at –1.05 V vs. RHE after CO_2RR in H-cells.

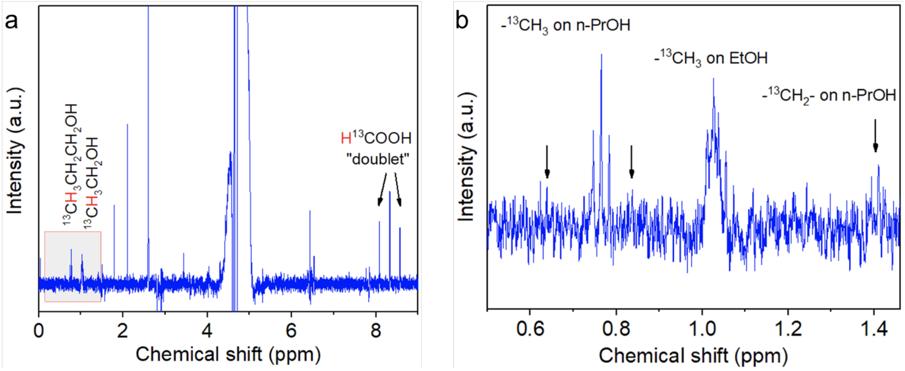

Supplementary Fig. 18 a ^1H NMR spectra of liquid products products from CuS_x-DSV catalyst at –1.05 V vs. RHE using $^{13}CO_2$ as feedstocks in H-cell. **b** The enlarged spectra (red rectangle in **a**) of $–^{13}CH_3$ and $–^{13}CH_2–$ groups n-PrOH, and $–^{13}CH_3$ group on ethanol.

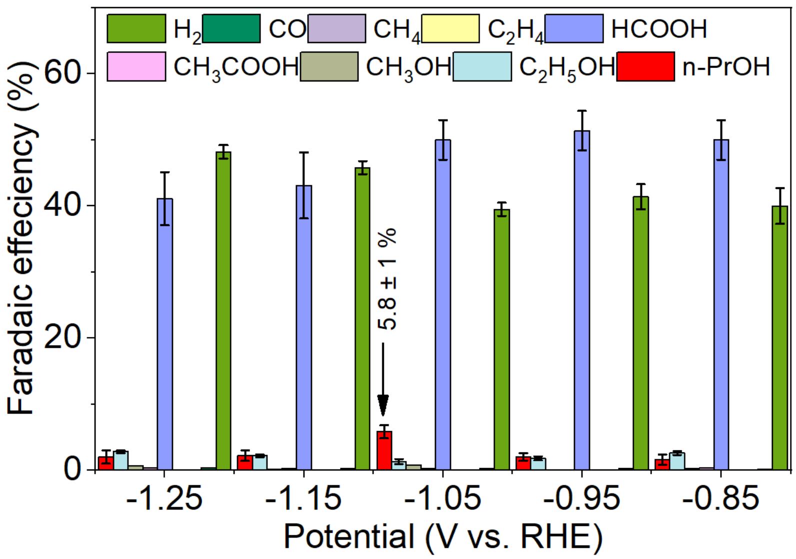

Supplementary Fig. 19 CO_2RR products distribution of CuS_x-1-cycle in H-cells. Error bars correspond to mean ± standard deviation at three measurements.

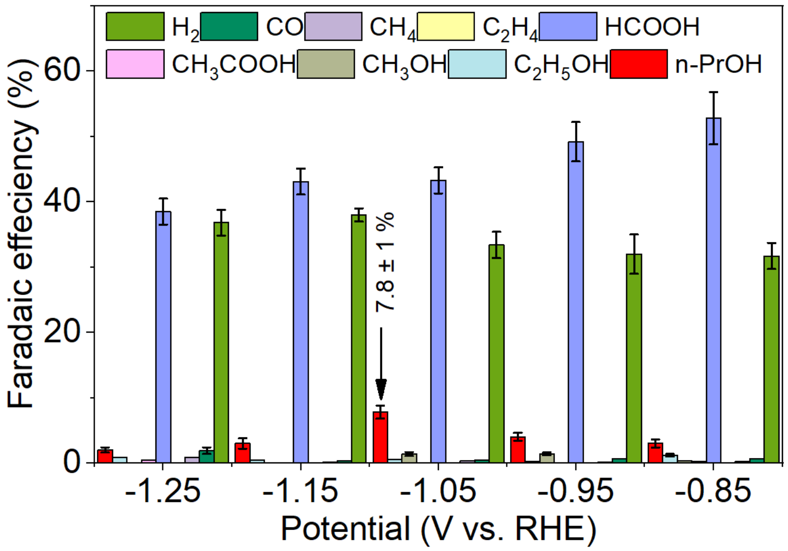

Supplementary Fig. 20 CO_2RR products distribution of CuS_x-100-cycle in H-cell. Error bars correspond to mean ± standard deviation at three measurements.

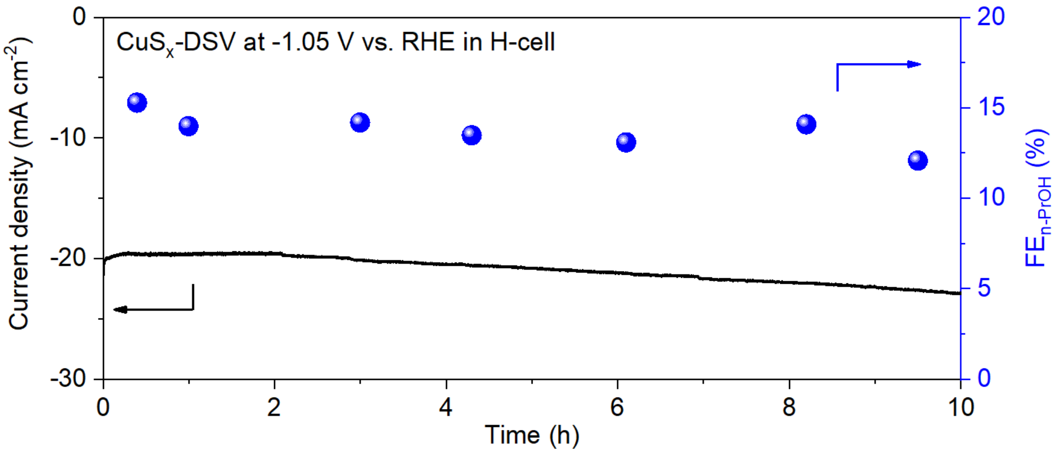

Supplementary Fig. 21 Chrono-amperometry results of CuS_x-DSV at a potential of –1.05 V versus RHE in a H-cell.

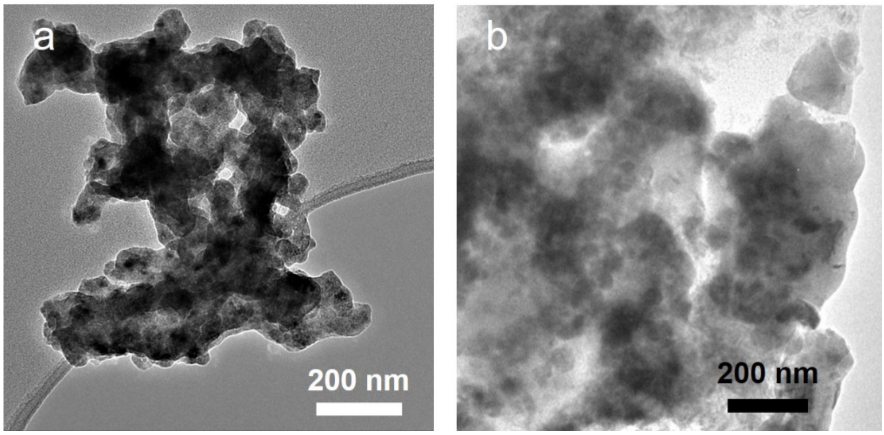

Supplementary Fig. 22 TEM images of **a** CuS and **b** CuS_x-DSV after CO_2RR at –1.05 V vs. RHE in H-cells.

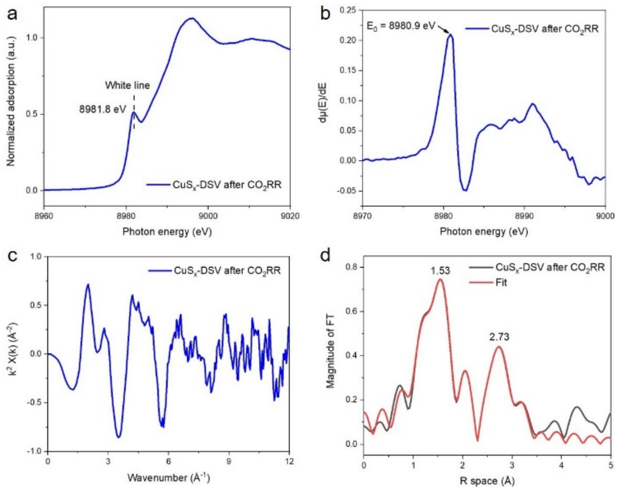

Supplementary Fig. 23 **a** Normalized Cu *K*-edge XANES spectra, **b** First derivative μ(E)/dE, **c** K-space and **d** Fourier-transformed $k^2\chi(k)$ of CuS_x-DSV catalyst after CO_2RR.

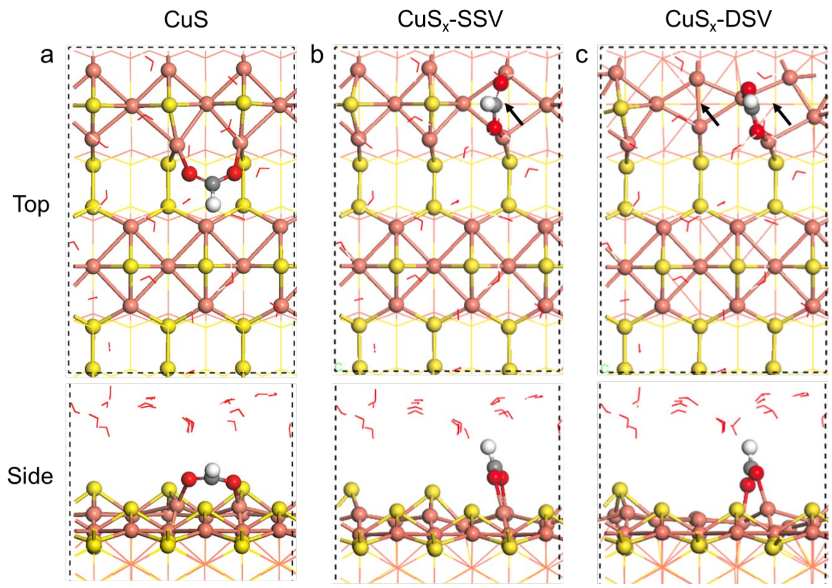

Supplementary Fig. 24 Top view and side view of the optimized *OCHO intermediate adsorbed on the (100) facets of **a** CuS, **b** CuS_x-SSV and **c** CuS_x-DSV. The pink, yellow, gray, red, white spheres, and red wireframes in **a**–**c** represent copper, sulfur, carbon, oxygen, hydrogen atoms, and water molecules, respectively. The arrows in **b** and **c** indicate the positions of sulfur vacancies.

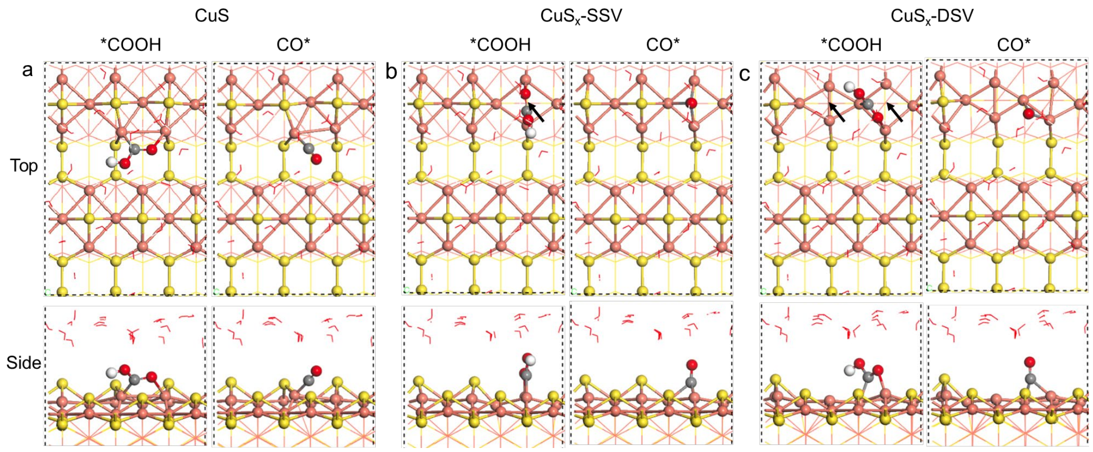

Supplementary Fig. 25 Top view and side view of the optimized *COOH and CO* intermediates adsorbed on the (100) facets of **a** CuS, **b** CuS_x-SSV and **c** CuS_x-DSV. The pink, yellow, gray, red, white spheres, and red wireframes in **a**–**c** represent copper, sulfur, carbon, oxygen, hydrogen atoms, and water molecules, respectively. The arrows in **b** and **c** indicate the positions of sulfur vacancies.

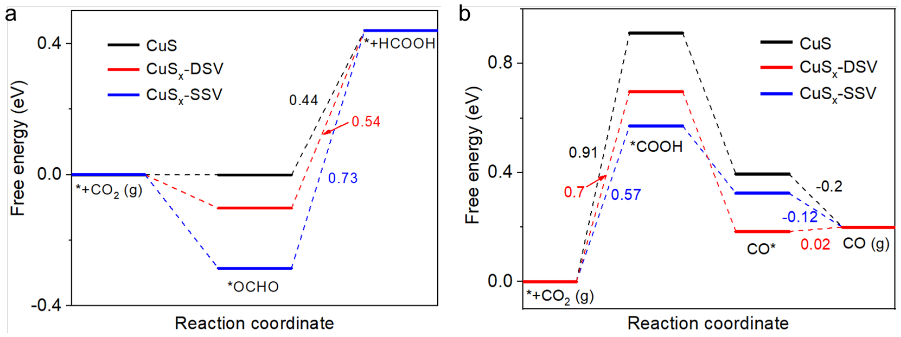

Supplementary Fig. 26 The free energy diagrams of HCOOH and CO formation at 0 V vs. RHE on the (100) facets of **a** CuS, **b** CuS_x-SSV and **c** CuS_x-DSV. Here it should be noted that the energy profile of C–C coupling (in **Fig. 1d**) cannot be directly compared with HCOOH formation. First, the C–C coupling step is a non-electrochemical step (no electron transfer), different from the electrochemical production of HCOOH or CO that can be affected by applied potential. Second, the energy barrier for C–C coupling is a simplified but reasonable descriptor to C_{2+} yields.

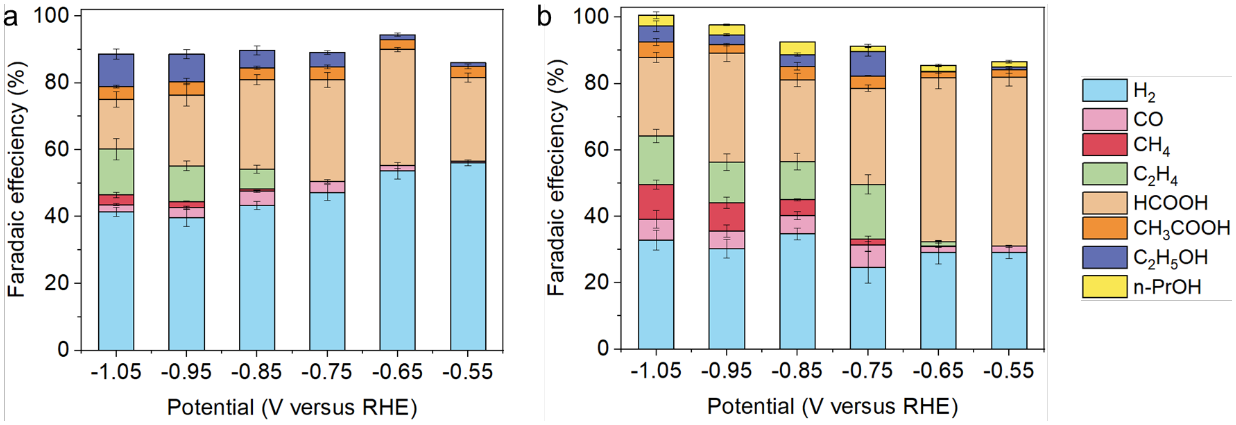

Supplementary Fig. 27 CO_2RR products distributions of **a** CuS and **b** CuS_x-DSV in flow-cells. Error bars in **a** and **b** correspond to mean ± standard deviation at three measurements.

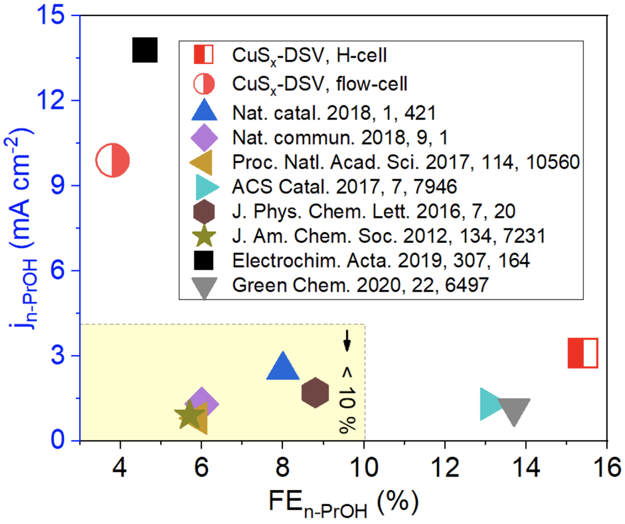

Supplementary Fig. 28 Comparison of electrochemical performances of $j_{n\text{-}PrOH}$ vs. $FE_{n\text{-}PrOH}$ during CO_2 reduction to n-propanol using Cu-based electrocatalysts.

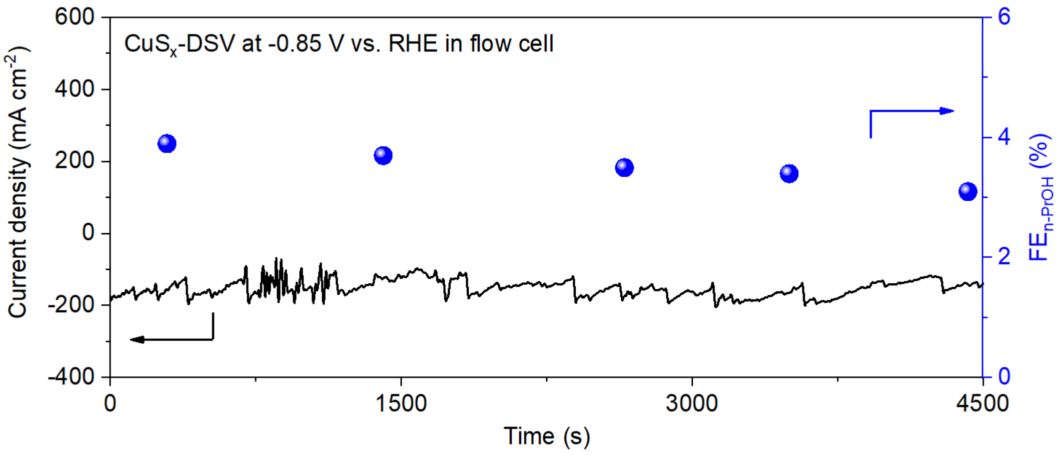

Supplementary Fig. 29 Chrono-amperometry results of CuS_x-DSV at a potential of –0.85 V versus RHE in a flow cell. The relative unstable current density in flow cell can be ascribed to the violent reaction condition caused by the electrolyte flooding and bubble evolution[7].

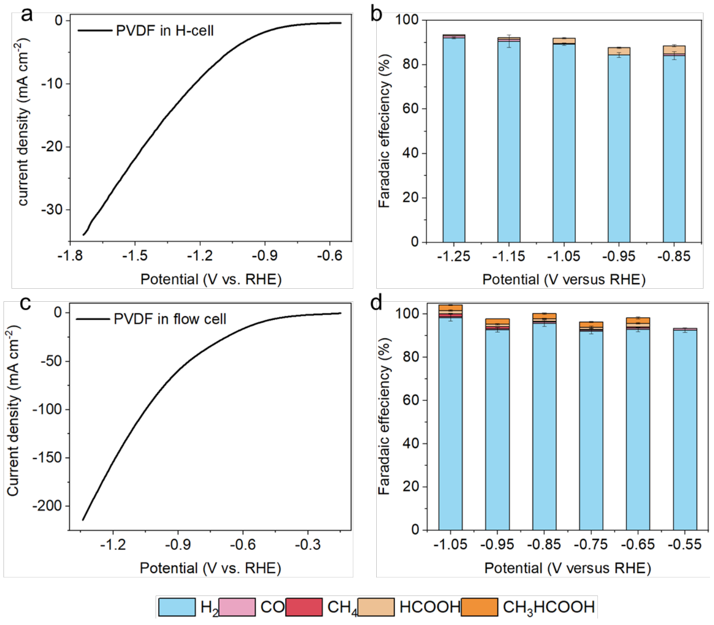

Supplementary Fig. 30 Linear sweep voltammetry curves and CO_2RR products distribution of PVDF binder catalyst in **a**, **b** H-cell and **c**, **d** flow cells. Error bars in **b** and **d** correspond to mean ± standard deviation at two measurements.

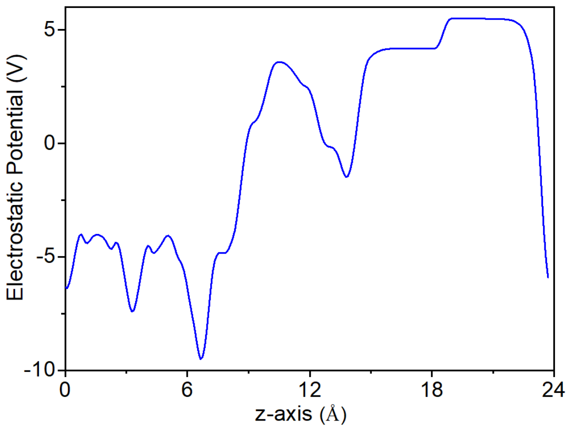

Supplementary Fig. 31 Plane-averaged electrostatic potential for a CuS (100) slab with one water layer on one side.

Supplementary Table 1 The shortest distance of Cu–Cu (denoted as DX, X=1, 2, 3 and 4) extracted from the final optimized model in **Supplementary Fig. 2**.

	Single	Double	Triple	Quadruple
D1 (Å)	3.259	3.540	3.367	3.378
D2 (Å)	3.481	3.825	3.700	3.469
D3 (Å)	3.811	4.178	3.810	3.590
D4 (Å)	3.947	4.208	3.825	3.836

For the model of single sulfur vacancy, the distance is extracted from the neighboring Cu atoms around the vacancy; for the model of double, triple and quadruple sulfur vacancies, the CO–CO coupling should take place at the two Cu atoms located at the different vacancy sites. Thus, the distance is extracted from the Cu atoms of the adjacent vacancy sites. Apparently, the shortest distance mostly varies from 3 Å to 4 Å.

Supplementary Table 2 Energy barriers and energy change of CO* dimerization and the following CO–OCCO coupling on the CuS (100) surface. CuS with single sulfur vacancy and double sulfur vacancies were calculated by the charged water model, respectively. All energies are in eV. The "+" or "–" represent the increment or decrement of energy.

		Energy barriers	Energy change
CuS_x-SSV	OCCO*	0.6	–0.24
	OCCOCO*	0.29	+0.1
CuS_x-DSV	OCCO*	0.2	–0.35
	OCCOCO*	0.33	–0.23

Supplementary Table 3 The distance of Cu–Cu extracted from the final optimized model in **Fig. 1b, Fig. 1c, Supplementary Fig. 3** and **Supplementary Fig. 5**.

	CuS_x-SSV *OCCOCO	CuS_x-DSV *OCCOCO
D1 (Å)	2.573	2.509
D2 (Å)	2.500	2.457
D3 (Å)	2.621	2.531
D4 (Å)	2.548	2.499
D5 (Å)	2.680	2.506
D6 (Å)	2.597	2.468
D7 (Å)	2.581	2.465
D8 (Å)	2.589	2.638
Average	2.586	2.509
The reason why SSV-CuS_x model cannot dimerize the CO–COCO is not the far distance (2.586>2.509 Å of DSV-CuS_x model), but the strong electrostatic repulsion between the three CO* due to the triangle CO–OCCO centered at the only one sulfur vacancy site, where the latter adopt the polyline bonding way with the help of the neighboring sulfur vacancy to free the force.		

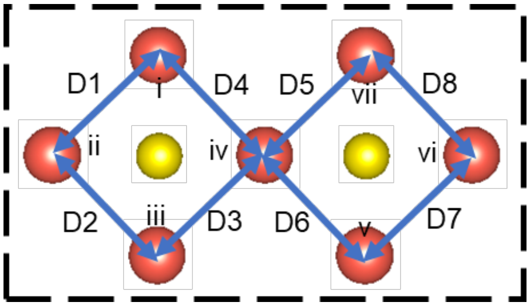

The attached illustration of basic sites constructed with seven Cu atoms (i→ vii) and two S atoms (or sulfur vacancies), as well as the definition of the measured distance extracted from the final optimized model in **Fig. 1b, Fig. 1c, Supplementary Fig. 3** and **Supplementary Fig. 5**.

Supplementary Table 4 EDS results of CuS, CuS_x-1-cycle, CuS_x-DSV and CuS_x-100-cycle in **Fig. 2b**.

	Elements	wt%	atomic%	S/Cu
CuS	Cu	62.07	45.23	1.21
	S	37.93	54.77	
CuS_x-1-cycle	Cu	68.03	51.78	0.92
	S	31.97	48.22	
CuS_x-DSV	Cu	74.60	59.71	0.67
	S	25.40	40.29	
CuS_x-100-cycle	Cu	76.19	60.98	0.64
	S	23.81	39.02	

Supplementary Table 5 EXAFS curve-fitting results of CuS nanosheets and CuS_x-DSV.

Sample	Path	CN	R (Å)	σ^2 (10^{-3} Å^2)	ΔE (eV)	S_0^2	R-factor
CuS theory	Cu–S	3	2.30				
	Cu–Cu	3	3.21				
CuS nanosheets	Cu–S	3	2.26±0.01	5.3±0.9	5.7±0.5	0.71±0.03	0.001
	Cu–Cu	3	3.23±0.03	25.6±5.0	5.7±0.5	0.71±0.03	0.001
CuS_x-DSV	Cu–S	1.0±0.2	2.32±0.02	4.8±2.2	9.0±2.0	0.90	0.017
	Cu–Cu	1.1±0.8	2.78±0.02	13.5±8.8	9.0±2.0	0.90	0.017

During the fitting of CuS nanosheets, its coordination numbers was fixed as the same values as that of CuS theory, while the internal atomic distances R, Debye–Waller factor σ^2, the edge-energy shift ΔE and passive electron reduction factor S_0^2 were allowed to run freely. During the fitting of CuS-10 cycles, S_0^2 was fixed to be the value of 0.9, and the other four parameters were variable. The error bar in the fitting results for these samples were provided.

Supplementary Table 6 The $FE_{n\text{-}PrOH}$ values with the sulfur vacancies concentration calculated based on the double sulfur vacancies/total sulfur atoms in the DFT model in **Fig. 2b**, and the fitting coordination number obtained from the EXAFS in **Supplementary Table 5**.

<table>
<tr><th rowspan="2"></th><th colspan="2">Double sulfur vacancies concentration</th><th rowspan="2">$FE_{n\text{-}PrOH}$ (%)</th></tr>
<tr><th>DFT</th><th>EXAFS</th></tr>
<tr><td>CuS</td><td>0</td><td>0</td><td>2.3</td></tr>
<tr><td>CuS_x-DSV</td><td>2.53%</td><td>2.78%</td><td>15.4</td></tr>
<tr><td colspan="4">According to Supplementary Figure 1, in a single CuS cell, there are six inner copper atoms (occupying 100%) and three kinds of sulfur atoms in the upper or lower layers. The three kinds of sulfur atoms are two inner sulfur atoms (occupying 100%), sixteen sulfur atoms located on the rhombus edge (occupying 1/6), four extended sulfur atoms which are parallel to the plane of an axis or b axis (occupying 1/12).
Based on the EXAFS result, the fitting coordination number for CuS_x-DSV was 1.0±0.2, suggesting the missing of four extended sulfur atoms that are parallel to the plane of an axis or b axis on the upper and lower layers. Thus, the double sulfur vacancies concertation is calculated to be 2.78%, closed to the model of DFT calculations.</td></tr>
</table>

Supplementary Table 7 CO_2 reduction products at different applied potentials on CuS nanosheets electrocatalyst in H-cell system.

Potential (vs. RHE)	H_2	CO	CH_4	HCOOH	CH_3COOH	CH_3OH	C_2H_5OH	n-PrOH
–0.85	33.3±2.9	0.2	0	59.6±7.4	0.5	0.8	1.2±0.8	1.1±0.3
–0.95	29±0.9	0.2	0	60.1±7	0.2	0.1	1.1±0.5	1±0.2
–1.00	32.2±0.6	0.5	0	60.3±2.1	0.1	0.2	0.7	0.8
–1.05	30.6±0.9	0.1	0	58±2	0.3	0.1	1.1±0.3	2.3±0.9
–1.10	31.5±1	0.6	0	60.3±1.9	0.1	0.1	0.9	0.8
–1.15	26.7±2.7	0.2	0.1	60.2±4.6	0.1	0.1	1.5±0.3	1±0.3
–1.25	32±2.2	0.3	0.1	54.3±0.8	0.2	0	0.1	1.1±0.5

Supplementary Table 8 CO_2 reduction products at different applied potentials on CuS_x-DSV electrocatalyst in H-cell system.

Potential (vs. RHE)	H_2	CO	CH_4	HCOOH	CH_3COOH	CH_3OH	C_2H_5OH	n-PrOH
–0.85	35.6±2.7	0.1	0.2	44.4±4.5	0.3	0.5	1.5±1.4	5.7±0.8
–0.95	28.2±1.9	0.4	0.1	47.6±3.0	0.3	0.3	1.4±0.7	6.0±0.6
–1.00	22.4±2.5	1.1	0.1	50.7±2.5	0.6	0.1	2.3±1.3	10.5±1.5
–1.05	21.2±1	1	0.2	48.8±0.9	0.6	0.8	2.4±1.5	15.4±1
–1.10	22.2±2.1	0.9	0.2	47.5±1.9	0.4	0	1.2±0.5	9.0±1.6
–1.15	28.7±1	0.7	0.1	42.5±5.0	0.1	0.5	3.1±1.5	5.4±0.8
–1.25	37.2±1	1.4±0.3	1.1	44.4±3.9	0.2	0.1	3.6±2.4	2.1±1

Supplementary Table 9 EXAFS curve-fitting results of CuS_x-DSV after CO_2RR.

Path	CN	R (Å)	σ^2 (10^{-3} Å^2)	ΔE (eV)	S_0^2	R-factor
Cu–S	0.6	2.19	9.3	4.9	0.9	0.011
Cu–Cu	4.2	3.08	30	4.9	0.9	0.011

Supplementary Table 10 CO_2 reduction products at different applied potentials on CuS nanosheets electrocatalyst in flow cell.

Potential (vs. RHE)	H_2	CO	CH_4	C_2H_4	HCOOH	CH_3COOH	C_2H_5OH	n-PrOH
–0.55	56.1±0.8	0.4	0	0	25.1±1.3	3.3±0.7	1.1±0.1	0
–0.65	53.7±2.5	1.6±0.8	0	0	34.7±0.6	2.9±0.1	1.5±0.5	0
–0.75	47.2±2.4	3.3±0.5	0	0	30.4±2.2	3.9±0.6	4.3±0.6	0
–0.85	43.3±1.2	4.3±0.3	0.7	5.9±1.2	26.7±1.5	3.6±0.5	5.2±1.3	0
–0.95	39.6±2.5	3.1±0.4	1.8±0.2	10.7±1.4	21.2±3.3	4±1	8.2±1.3	0
–1.05	41.4±1.4	2.2±0.3	2.9±0.8	13.7±3.2	14.8±2.3	3.9±0.4	9.7±1.5	0

Supplementary Table 11 CO_2 reduction products at different applied potentials on CuS_x-DSV electrocatalyst in flow cells.

Potential (vs. RHE)	H_2	CO	CH_4	C_2H_4	HCOOH	CH_3COOH	C_2H_5OH	n-PrOH
–0.55	29.2±2	1.8±0.3	0	0	50.9±2.6	2.3±1	0.8	1.6±0.4
–0.65	29.1±3.5	1.8±0.2	0.1	1.3±0.4	49.4±3.3	1.8±0.3	0.2	1.8±0.3
–0.75	24.6±4.7	6.7±1.7	1.9±0.8	16.4±2.9	29±1	3.7±0.1	7.3±1.4	1.7±0.5
–0.85	34.7±1.8	5.5±1.2	4.8±0.3	11.5±2.5	24.6±2	4.1±1.1	3.5±0.5	3.8±0.1
–0.95	30.2±2.8	5.4±1.8	8.5±1.7	12.3±2.5	32.7±2.5	2.6±0.4	3±0.3	3±0.2
–1.05	32.8±3	6.3±2.7	10.5±1.4	14.6±2	23.7±1.5	4.6±1	4.8±1.6	3.3±1.1

Supplementary Table 12 Electrochemical performance summary of CO_2 reduction to n-propanol using Cu-based electrocatalysts.

Electrocatalysts	Types of cell	Electrolytes	Applied constant potential (vs. RHE)	FE and j (mA cm^{-2}) /n-PrOH	References
CuS_x-DSV	H-cell	0.1 M $KHCO_3$	–1.05 V	15.4%/3.1	This work
CuS_x-DSV	Flow-cell	1 M KOH	–0.85 V	3.8%/9.9	This work
Cu_2S-Cu-V	H-cell	0.1 M $KHCO_3$	–0.95 V	8%/2.5	Nat. Catal. 2018, 1, 421
Cu-on-Cu_3N	H-cell	0.1 M $KHCO_3$	–0.95 V	6%/1.3	Nat. Commun. 2018, 9, 1
Cu nanoparticles	H-cell	0.1 M $KHCO_3$	–0.81 V	5.9%/0.8	Proc. Natl. Acad. Sci. 2017, 114, 10560
Activated Cu mesh	H-cell	0.5 M $KHCO_3$	–0.9 V	13.1%/1.3	ACS Catal. 2017, 7, 7946
Agglomerated Cu nanocrystals	H-cell	0.1 M $KHCO_3$	–0.95 V	8.8%/1.7	J. Phys. Chem. Lett. 2016, 7, 20
Cu_2O-derived	H-cell	0.5 M $NaHCO_3$	–0.85 V	5.7%/0.9	J. Am. Chem. Soc. 2012, 134, 7231
oxide-derived Pd_9Cu_{91}	H-cell	0.5 M $KHCO_3$	–0.65 V	13.7%/1.15	Green Chem. 2020, 22, 6497
Cu-nanoparticles	Flow-cell	1 M $KHCO_3$	–0.97 V	4.6%/13.8	Electrochim. Acta. 2019, 307, 164

Supplementary References

1. Perdew, J.P. *et al.* Generalized gradient approximation made simple. *Phys. Rev. Lett.* **77**, 3865-3868 (1996).
2. Kresse, G. *et al.* Efficiency of ab-initio total energy calculations for metals and semiconductors using a plane-wave basis set. *Comp. Mater. Sci.* **6**, 15-50 (1996).
3. Skúlason E. et al. Modeling the electrochemical hydrogen oxidation and evolution reactions on the basis of density functional theory calculations. *J. Phys. Chem. Lett.* **114**, 18182-18197 (2010).
4. Chen, L. et al. Electric field effects in electrochemical CO_2 reduction. *ACS Catal.* **6**, 7133-7139 (2016).
5. Grimme, S. *et al.* A consistent and accurate ab initio parametrization of density functional dispersion correction (DFT-D) for the 94 elements H-Pu. *J. Chem. Phys.* **132** (2010).
6. Henkelman, G. *et al.* A climbing image nudged elastic band method for finding saddle points and minimum energy paths. *J. Chem. Phys.* **113**, 9901-9904 (2000).
7. Lv, J. *et al.* A highly porous copper electrocatalyst for carbon dioxide reduction. *Adv. Mater.* **30**, 1803111 (2018).
